# Burn resuscitation strategy influences the gut microbiota-liver axis in swine

**DOI:** 10.1038/s41598-020-72511-8

**Published:** 2020-09-24

**Authors:** Wayne T. Muraoka, Jose C. Granados, Belinda I. Gomez, Susannah E. Nicholson, Kevin K. Chung, Jeffrey W. Shupp, James A. Bynum, Michael A. Dubick, David M. Burmeister

**Affiliations:** 1 Blood Coagulation Research Department, USA Army Institute of Surgical Research, JBSA Ft. Sam Houston, San Antonio, TX 78234 USA; 2 Division of Trauma and Emergency Surgery, Department of Surgery, University of Texas Health Science Center, San Antonio, TX 78229 USA; 3 Department of Medicine, Uniformed Services University of the Health Sciences, 4301 Jones Bridge Road, Building 42, Room 3129, Bethesda, MD 20814 USA; 4 The Burn Center, MedStar Washington Hospital Center, Department of Surgery, Georgetown University School of Medicine, Washington, DC 20010 USA

**Keywords:** Microbiome, Trauma, Experimental models of disease

## Abstract

Fluid resuscitation improves clinical outcomes of burn patients; however, its execution in resource-poor environments may have to be amended with limited-volume strategies. Liver dysfunction is common in burn patients and gut dysbiosis is an understudied aspect of burn sequelae. Here, the swine gut microbiota and liver transcripts were investigated to determine the impact of standard-of-care modified Brooke (MB), limited-volume colloid (LV-Co), and limited-volume crystalloid (LV-Cr) resuscitation on the gut microbiota, and to evaluate its' potential relationship with liver dysfunction. Independent of resuscitation strategy, bacterial diversity was reduced 24 h post-injury, and remained perturbed at 48 h. Changes in community structure were most pronounced with LV-Co, and correlated with biomarkers of hepatocellular damage. Hierarchical clustering revealed a group of samples that was suggestive of dysbiosis, and LV-Co increased the risk of association with this group. Compared with MB, LV-Co and LV-Cr significantly altered cellular stress and ATP pathways, and gene expression of these perturbed pathways was correlated with major dysbiosis-associated bacteria. Taken together, LV-Co resuscitation exacerbated the loss of bacterial diversity and increased the risk of dysbiosis. Moreover, we present evidence of a linkage between liver (dys)function and the gut microbiota in the acute setting of burn injury.

## Introduction

Burn injuries are the fourth leading cause of traumatic injury in civilian populations and account for over 300,000 global deaths each year^1^. Within the military population, 9–13% of in-hospital deaths were attributed to burns^2,3^. Large burn injuries involving > 20% of the total body surface area (TBSA) cause profound physiological changes that extend beyond the site of injury, and it is well recognized that liver and intestinal functions are important contributors to patient outcomes^4–6^. Clinical and preclinical studies demonstrated that burn injury reduces liver and intestinal blood flow, promotes hepatomegaly, and increases intestinal permeability and bacterial translocation^7–12^. Indeed, over half of the patients that succumbed to their thermal injuries exhibit ischemic necrosis of the intestine with high prevalence of sepsis^13^. The gut and liver communicate via the hepatobiliary, portal, and systemic circulatory systems, and recent evidence suggests that the gut microbiota influence the gut-liver axis in chronic diseases^14^. However, whether this relationship plays a role after traumatic injury, such as burns, remains to be addressed.

Emerging evidence suggests that the intestinal microbiota can become dysregulated early after acute traumatic injury^15–18^, and that bacterial diversity is reduced post-burn^8^. Consistent with these clinical findings, animal models have revealed that burn injury reduces bacterial diversity, and increases the abundance of Proteobacteria^10,19,20^. Thus, gut dysbiosis may be an overlooked aspect of burn sequelae and the impact of medical intervention on the gut microbiota is poorly understood. For example, antibiotics and fluid resuscitation are aspects of patient care that can have downstream effects on the microbiome and other outcomes.

Patients with large burns receive early and aggressive intravenous (IV) fluid resuscitation to restore intravascular volume and maintain organ perfusion^21^. While both crystalloids (e.g. Lactated Ringer’s [LR] and PlasmaLyte [PL]) and colloids (e.g. fresh frozen plasma [FFP] and albumin [AB]) have been used to achieve this, there is not unanimous consensus on the best strategy within the burn community. Under-resuscitation is inherently dangerous, while over-resuscitation can put the patient at risk of edema and compartment syndromes^22^. In resource poor environments (e.g., civilian mass casualty scenarios or delayed transport), limited-volume resuscitation may achieve this goal, and may have an effect on various physiological outcomes^23,24^. In the present study, we compared standard-of-care resuscitation to limited-volumes of colloids and crystalloids with an aim to evaluate the impact of different resuscitation strategies on the gut microbiota and hepatic (dys)function and gene expression.

## Results

### Effects of burn resuscitation on pathophysiology

**Figure 1:**
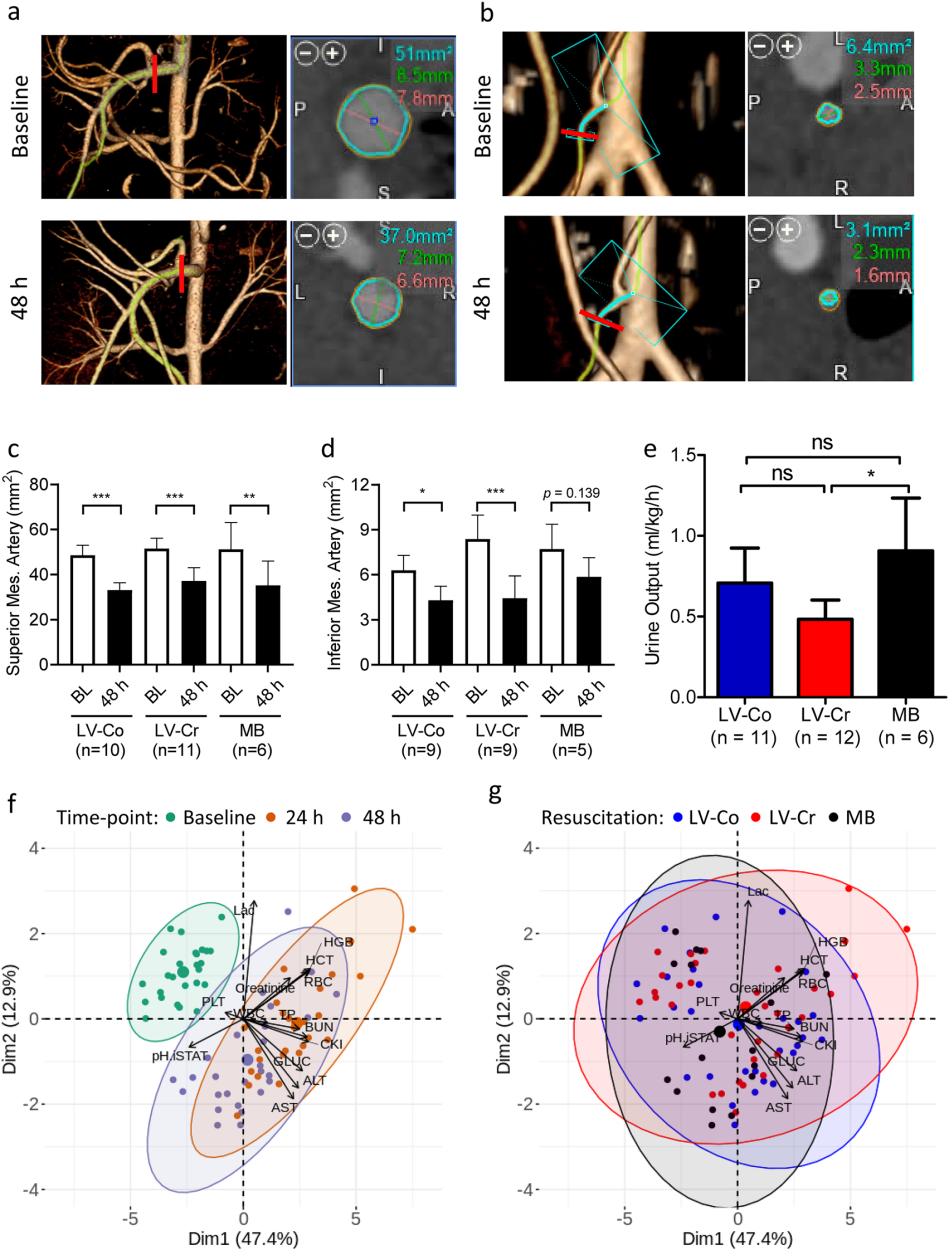
Limited-volume resuscitation does not exacerbate measured physiologic derangements compared with MB. (**a, b**) Representative CT angiogram of the (**a**) superior mesenteric artery (SMA) and (**b**) inferior mesenteric artery (IMA). Left panel: green trace indicates selected vessel with red bars indicating the location of cross-sectional measurements. Right panel: cross-section of the selected vessel with blue circle outlining the vessel lumen; red and green lines depict two measurements of vessel lumen diameter. Text indicates measurements of area (blue) and diameter (red and green). Images were acquired with the Vitrea software v6.9.87.1 (https://www.vitalimages.com/vitrea/). (**c, d**) Quantification of cross-sectional areas of (**c**) SMA and (**d**) IMA at baseline (BL) and 48 h post-injury. Bars depict mean and 95% confidence intervals. Images that did not show sufficient contrast could not be measured accurately and were excluded from analysis. (**e**) Urine output 20 h after burn injury. Experimental replicates are indicated in each respective figure. (**f, g**) Dimension reduction analysis of 14 clinical biomarkers visualized by PCA biplots with (**f**) time post-injury or (**g**) resuscitation strategy supplied as supplementary variables. Each small dot depicts an individual sample, larger dots indicate the centroid of the ellipse. Ellipses depict 95% confidence region. MB (n = 6 pigs), LV-Co (n = 12 pigs), and LV-Cr (n = 12 pigs) *: *p* < 0.05; **: *p* < 0.01; ***: *p* < 0.001, ns: *p* > 0.05 calculated by ANOVA with Holm family-wise error rate correction at α = 0.05.

We measured the cross-sectional area of the superior (SMA) and inferior (IMA) mesenteric arteries from computed tomography (CT) angiograms (Fig. 1a,b) as a proxy for intestinal perfusion. The cross-sectional areas for both vessels were significantly reduced at 48 h post-burn compared with baseline (Fig. 1c,d); however, both SMA [F(2,24) = 0.86, *p* = 0.44] and IMA [F(2,24) = 2.85, *p* = 0.08] were not significantly different across resuscitation strategies. To evaluate the extent of fluid shift from the intravascular space, we measured total urine output and edema in both burned and non-burned tissues 48 h post-injury. Normalized urine output was higher in the MB group compared to LV-Cr, but LV-Co was not different from either group (Fig. 1e). We also measured edema in burn-injured and uninjured skin via wet-to-dry weight ratios (Supplementary Fig. S1). Uninjured skin had less edema than burn-injured skin [F(1,26) = 20.10, *p* < 0.01], but resuscitation strategy did not significantly influence edema [F(2,27) = 0.67, *p* = 0.52].

We next evaluated a panel of 14 clinical biomarkers that are routinely used as diagnostic and prognostic indicators of shock and organ function (Supplementary Table S1). Analysis of these markers revealed acidosis (pH, lactate), hemoconcentration of blood components (red blood cell count, hematocrit, hemoglobin), hyperglycemia, and liver and kidney dysfunction (aspartate and alanine aminotransferases [AST and ALT, respectively], creatinine, urea nitrogen). Dimension reduction analysis with PCA plots revealed alterations in these biomarkers after burn injury (Fig. 1f), but not across resuscitation strategies (Fig. 1g). Not surprisingly, red blood cell count, hematocrit, and hemoglobin were positively correlated, all of which negatively correlated with pH. Lactate was uncorrelated or weakly correlated to urea nitrogen, creatine kinase, and total protein. All associations were confirmed by linear regression (Supplementary Fig. S1). The biomarkers that characterized post-injury time points from baseline were AST, ALT, glucose, and to a lesser extent total protein, urea nitrogen, and creatine kinase (Fig. 1f). Variables that distinguished 24 h and 48 h post-injury time points were hemoglobin, hematocrit, red blood cell count, pH, and to a lesser extent creatinine. Further stratification of the dataset to visualize each combination of time and resuscitation did not reveal additional insight beyond separation by post-injury time (Supplementary Fig. S1). The effect of resuscitation across injury time was significant only for total protein [F(4,53) = 13.07, *p* < 0.01] with MB significantly different than LV-Co at 48 h (Tukey’s *p* < 0.01).

To evaluate the effects of burn and resuscitation on systemic inflammation, we measured plasma cytokines (Supplementary Fig. S2). IL-1ra was significantly elevated 48 h post-burn for both limited-volume strategies (Holm adjust *p* = 0.03), and IL-6 was significantly increased 48 h post-burn with LV-Co resuscitation (Holm adjusted *p* = 0.006). Cytokine levels within a particular time-point did not differ between resuscitation strategies. Finally, we evaluated the impact of resuscitation on intestinal villi architecture and mucosal inflammation by histology (Supplementary Fig. S3). The intestinal villi of all pigs appeared with minimal loss of goblet cells and moderate blunting that was not different amongst the groups. LV-Co tended to have greater inflammation than LV-Cr and MB; however these differences were not statistically significant (*p* = 0.78 and 0.21, respectively). Together, these findings suggest that, compared with standard-of-care resuscitation, LV-Co and LV-Cr did not exacerbate measured physiological derangements, fluid shift from the vascular space, or histological changes in the gut after burn injury. However, plasma IL-6 was significantly elevated after burn in the LV-Co group.

### Altered bacterial diversity is more pronounced with LV-Co resuscitation

**Figure 2:**
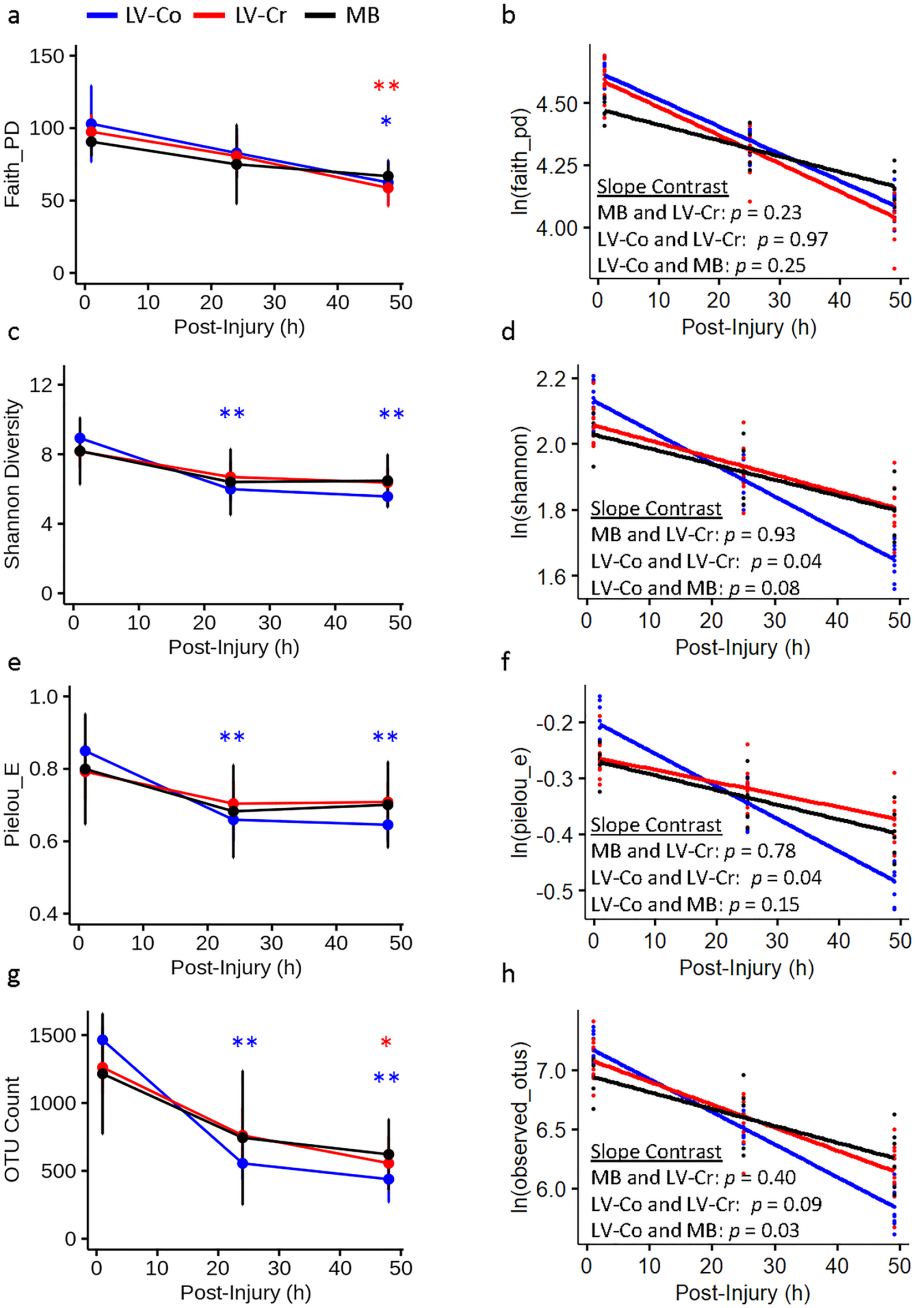
Impact of resuscitation on bacteria alpha diversity. (**a, b**) Faith phylogenetic diversity, (**c, d**) Shannon diversity, (**e, f**) Pielou evenness, and (**g, h**) OTU counts. (**a, c, e, g**) Raw diversity values with each point depicting the mean and bars representing 95% CI. Within treatment differences between post-injury (24 h and 48 h) and respective baseline time-points were evaluated with two-sided Wilcoxon rank sum test with Holm family-wise error rate correction at α = 0.05. Statistical significance is indicated by text and symbol colors corresponding to the resuscitation strategy. *: *p* < 0.05; **: *p* < 0.01 with actual *p* values indicated in text. (**b, d, f, h**) LME model predicted values and regression line after normalization of the data. Each data point depicts the predicted value of an individual sample. Comparison of regression slopes and *p* value calculations were evaluated by LME models. MB (n = 6 pigs), LV-Co (n = 10 pigs), and LV-Cr (n = 11 pigs).

Next, we evaluated the effect of resuscitation strategy on gut bacteria diversity. Of the 90 rectal swabs (30 pigs, three time-points), two LV-Co subjects, one LV-Cr subject, one 48 h LV-Co sample, and one baseline MB sample did not yield sufficient DNA and were excluded from further analysis. This yielded 79 samples which consisted of the following: MB (n = 6 pigs), LV-Co (n = 10 pigs), and LV-Cr (n = 11 pigs). The mean sequencing depth for each sample was 91,828 reads (range: 50,623–138,459). Taxonomic classification at the phylum level revealed heterogeneity among samples at baseline (Supplementary Fig. S4), providing insight into the variability of the healthy pig microbiota. To account for this variability, we focused our analysis on within-subject changes pre- (baseline) and post-injury (24 h and 48 h) using a repeated measures approach. Faith’s phylogenetic diversity was significantly lower for LV-Cr and LV-Co resuscitation at 48 h compared with pre-injury baseline samples (two-sided Wilcoxon *p* = 0.007 and *p* = 0.04, respectively; Fig. 2a). Bacterial diversity (Fig. 2c,e) was significantly reduced 24 h after injury and remained perturbed at 48 h post-injury for LV-Co (two-sided Wilcoxon *p* = 0.004). Bacterial richness (Fig. 2g) was reduced at 24 h and 48 h for LV-Co (two-sided Wilcoxon *p* = 0.004), and 48 h for LV-Cr (two-sided Wilcoxon *p* = 0.04). LME models revealed reduced α-diversity independent of resuscitation (negative slope coefficient with *p* < 0.01; Fig. 2b,d,f,h). The slope coefficients of LV-Co resuscitation differed significantly from LV-Cr for measurements of diversity (Fig. 2d,f; *p* = 0.04), and from MB for OTUs (Fig. 2h; *p* = 0.03).

**Figure 3:**
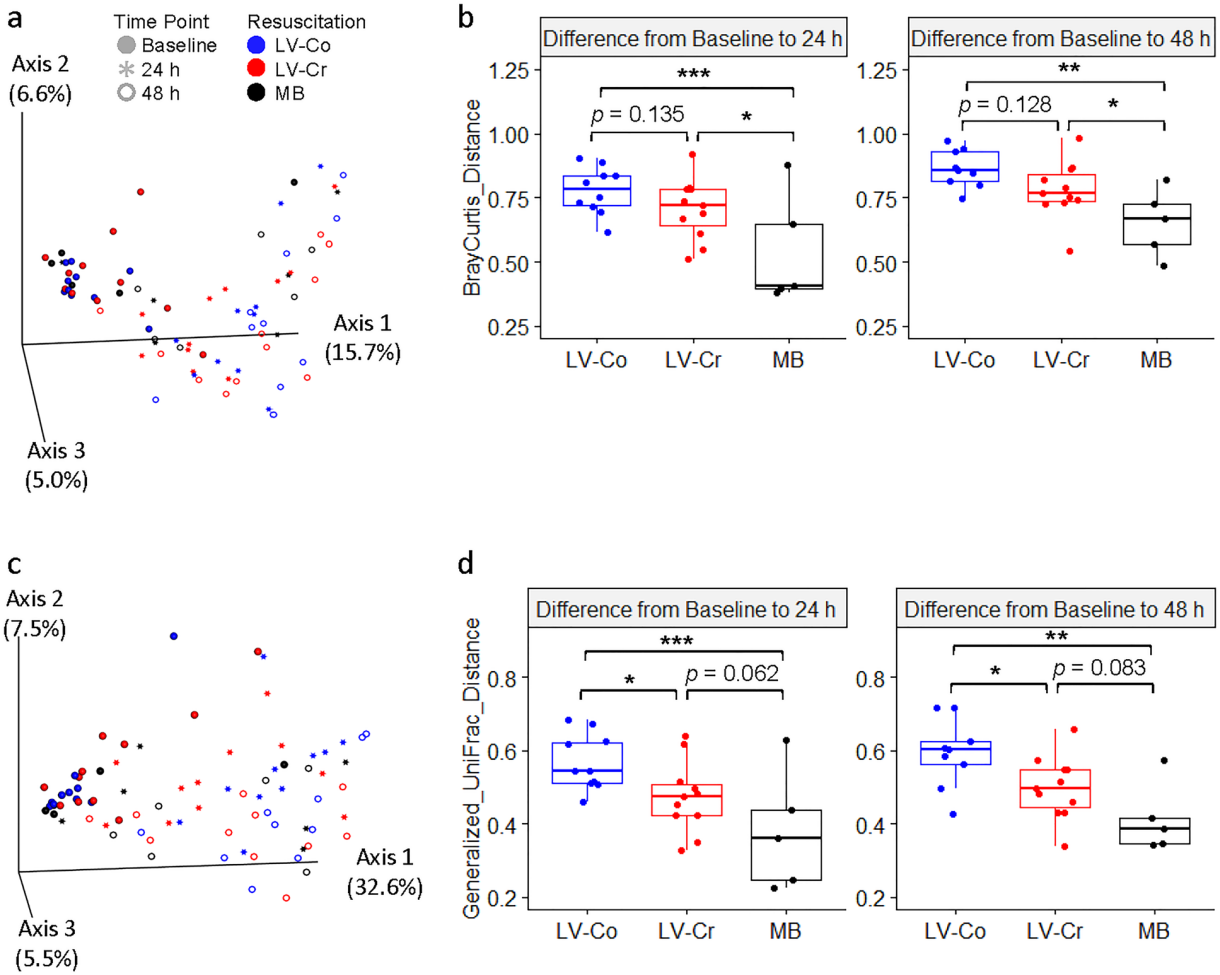
Impact of resuscitation on bacteria beta diversity. (**a**) Bray–Curtis and (**c**) generalized UniFrac distances visualized by PCoA plots. Symbol shapes depict sample time-points, whereas symbol colors represent different resuscitation strategies. Each data point represents an individual sample. The amount of variation explained by each axis are indicated in parentheses. Boxplots of (**b**) Bray–Curtis and (**d**) generalized UniFrac longitudinal first-distances. First-distances were calculated as the distance between post-injury (24 h and 48 h) time-points and its respective baseline. Each boxplot shows the median along with the first and third quartiles. Whiskers depict the standard error and data points in the boxplots represent an individual sample. *: *p* < 0.05; **: *p* < 0.01; ***: *p* < 0.001 calculated by LME models. MB (n = 6 pigs), LV-Co (n = 10 pigs), and LV-Cr (n = 11 pigs).

Visualization of β-diversity with Principal Coordinate Analysis (PCoA) plots revealed a shift in the microbiota within the first 24 h post-injury (Fig. 3a,c) with 27.3% and 45.6% of the total variance explained in Bray–Curtis and generalized UniFrac, respectively. There were slight, but significant, within-group dispersion differences for Bray–Curtis (Supplementary Fig. S5). We next evaluated whether resuscitation strategy affected the β-diversity rate of change by analyzing first-distances with LME models (Fig. 3b,d). First-distances were defined as the distance between each subject’s baseline and respective post-injury sample^25^. Whereas MB resuscitation yielded the smallest change from baseline, resuscitation with LV-Co produced the greatest change. MB resuscitation was significantly different from LV-Co for Bray–Curtis and generalized UniFrac at both post-injury time-points (Bray–Curtis: 24 h *p* < 0.001 and 48 h *p* = 0.003, UniFrac: 24 h *p* < 0.001 and 48 h *p* = 0.002). The differences between LV-Cr and MB were mixed with significant differences for only Bray–Curtis (24 h *p* = 0.01, 48 h *p* = 0.04). Together, these results suggest that LV-Co resuscitation is associated with greater loss of gut bacteria diversity.

### LV-Co resuscitation increases risk of dysbiosis

**Figure 4:**
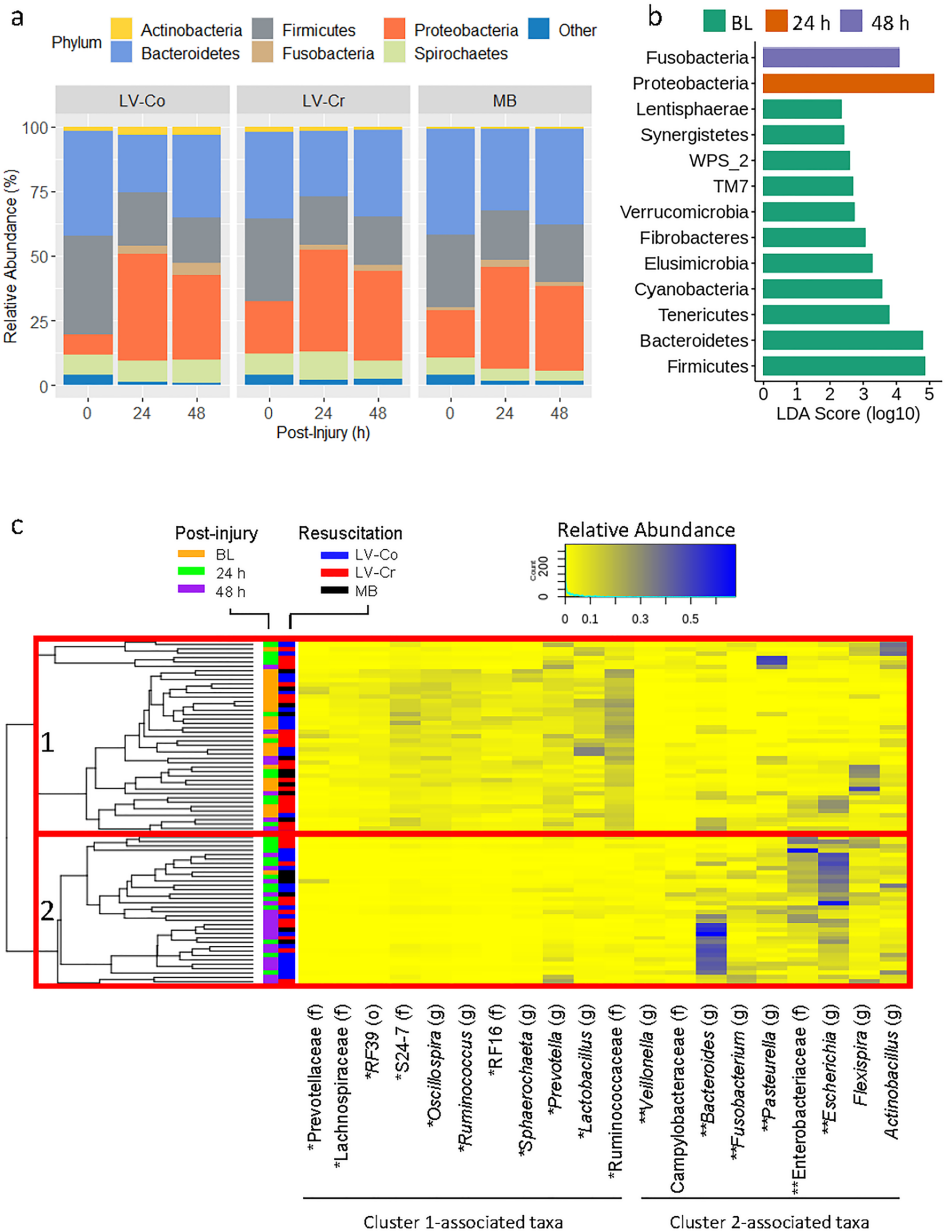
Altered bacterial composition after severe burn and resuscitation. (**a**) Stacked bar plot showing the mean relative abundance at the phylum level for LV-Co (n = 10 pigs), LV-Cr (n = 11 pigs), and MB (n = 5 pigs) resuscitation strategies. Only phyla consisting of ≥ 1% of the total bacterial community are shown; all other phyla are included in the “Other” category. (**b**) Differentially abundant phyla across time identified by LEfSe. Color of bars indicates enrichment of phyla at a particular time point. Only phyla with LDA score > 2 are shown. (**c**) Unsupervised clustering of the top 20 differentially abundant taxa scaled by relative abundance. Rows depict samples and columns indicate genus or the highest order taxon that could be classified (i.e., family or order). Letter in parentheses indicates the level of taxonomic classification: (o) order, (f) family, (g) genus. Compositional similarity of samples was assessed by hierarchical clustering. Clusters 1 and 2 are outlined by red rectangles. Each tip of the dendrogram represents an individual sample that is identified by injury time-point (orange, green, purple) and resuscitation (red, black, blue) side colors. Symbol preceding the taxa names indicate significant enrichment (Holm corrected two-sided Wilcoxon *p* < 0.05) in cluster 1(*) or cluster 2 (**).

Independent of resuscitation strategy, Firmicutes was reduced and Proteobacteria and Fusobacteria were increased after injury (Fig. 4a), with Bacteroides also transiently reduced after injury. Consistent with these trends, LEfSe identified Proteobacteria and Fusobacteria as more abundant in post-injury samples (Fig. 4b), whereas all other phyla were more abundant in baseline samples. However, many of the phyla enriched in baseline samples constituted < 1% of the total classified reads.

At the genus level, LEfSE identified 77 genera that were differentially abundant between baseline and post-injury time points (Supplementary Table S2), and 18 genera that were differentially abundant among MB, LV-Co, and LV-Cr resuscitation strategies (Supplementary Table S3). Several species and genera that are potentially pathogenic were enriched after burn injury (i.e., *Escherichia coli*, *Enterococcus* sp., *Pasteurella* sp., *Fusobacterium* sp., and *Staphylococcus aureus*). We next analyzed the 20 most abundant taxa (by rarefied sequence count) that differed significantly between pre- and post-injury samples by hierarchical clustering (Fig. 4c). Sample clustering based on compositional similarity revealed two major clusters, designated cluster 1 and cluster 2. Bootstrapping indicated that both clusters were highly stable with Jaccard similarity values of 0.85 and 0.83 for clusters 1 and 2, respectively.

Members of the order Clostridiales (i.e., unclassified genus of Lachnospiraceae, *Oscillospira*, unclassified genus of Ruminococcaceae, and *Ruminococcus*), Bacteroidales (i.e., S24-7, RF16, and *Prevotella*) and RF39, along with members of *Lactobacillus* and *Sphaerochaeta,* were significantly more abundant in cluster 1 samples (*p* < 0.05). In contrast, *Bacteroides*, an unclassified genus of Enterobacteriaceae, *Escherichia, Veillonella, Fusobacterium,* and *Pasteurella* were significantly more abundant in cluster 2 samples (*p* < 0.05). Notably, cluster 1 contained the preponderance of baseline samples (25/26; Binomial test *p* < 0.01) while cluster 2 contained the majority of samples from post-injury LV-Co subjects (16/20; Binomial test *p* < 0.01). Post-injury samples from LV-Cr and MB subjects were distributed with parity between cluster 1 and cluster 2 (Table 1). Taxa that were enriched with cluster 1 were greatly reduced in cluster 2, suggesting that samples in cluster 2 represented dysbiosis. Compared to MB and LV-Cr, LV-Co had 1.55 (95% CI 1.04 to 2.31, *p* = 0.04) times the risk of dysbiosis (association with cluster 2). To explore possible metabolic selection of dysbiosis-associated bacteria by LV-Co, we applied a computational approach to infer the functional metabolic capacity of the bacterial microbiota. However, we did not find any metabolic pathways that were differentially abundant among resuscitation strategies (ALDEx2 Kruskal–Wallis adjusted *p* > 0.05).

**Table 1:** Distribution of samples by hierarchical cluster, resuscitation, and sample time-point.

		LV-Co	LV-Cr	MB
Baseline	Cluster 1	10 (100)	11 (100)	4 (80)
	Cluster 2	0 (0)	0 (0)	1 (20)
Post-injury (24 h + 48 h)	Cluster 1	4 (20)	10 (48)	6 (50)
	Cluster 2	16 (80)^a^	11 (52)	6 (50)

Number of samples observed in cluster 1 and cluster 2 after stratifying by injury time and resuscitation. Numbers in parentheses indicate percentage of samples in each cluster.

^a^Risk of association with cluster 2 was 1.55 (95% CI 1.04 to 2.31, *p* = 0.04) times greater for LV-Co compared with MB and LV-Cr.

### Liver gene expression reflects dysbiosis after burn injury

**Figure 5:**
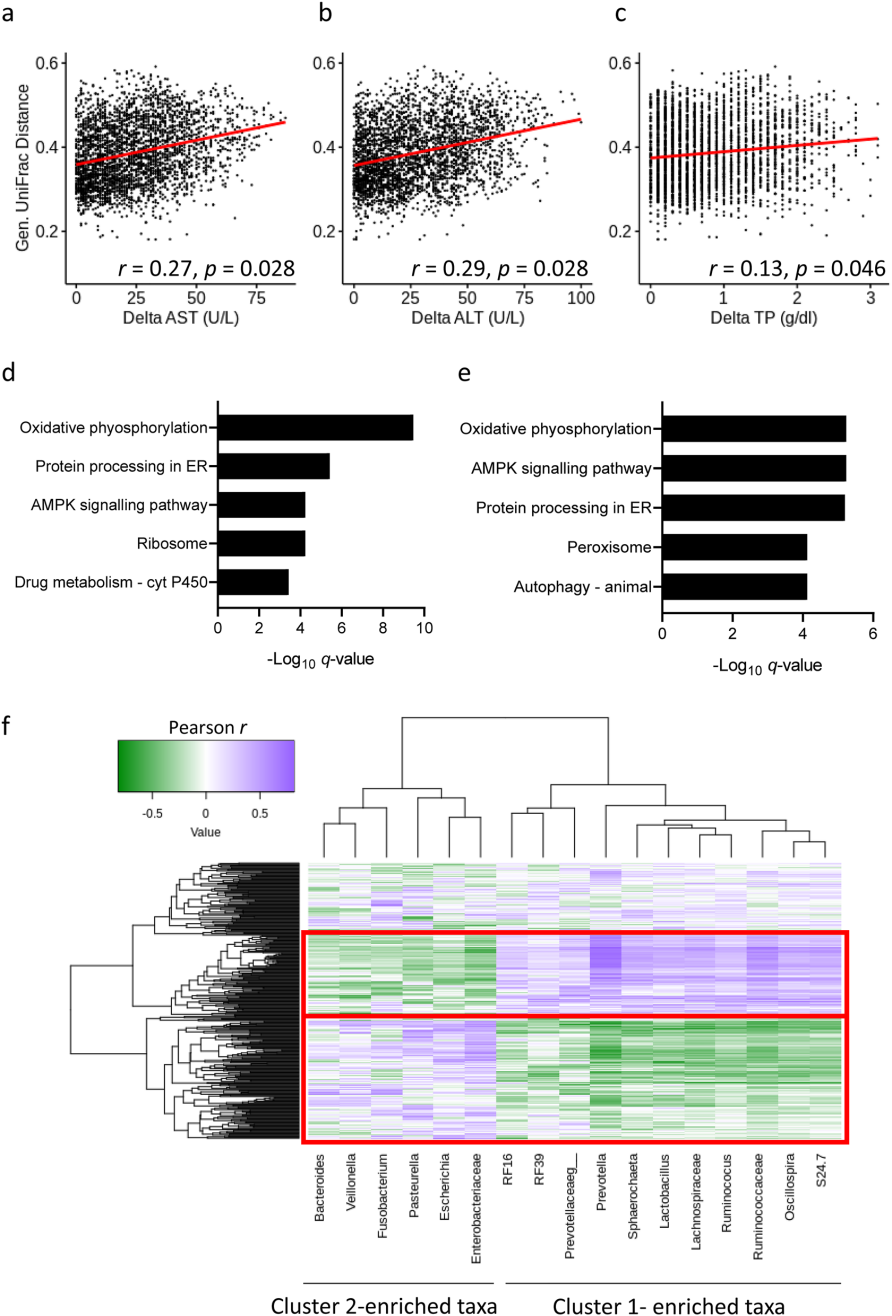
Changes in the bacterial diversity are associated with liver (dys)function. (**a–c**) Correlation between generalized UniFrac and (**a**) AST, (**b**) ALT, and (**c**) TP. Correlation coefficients were calculated by Mantel test and indicated *p* values were corrected using the Holm procedure for family-wise error rate at α = 0.05. Correlations between Bray–Curtis and AST, ALT, and total protein (TP) were similar (Supplementary Fig. S6). (**d, e**) Five most significantly perturbed pathways for (**d**) LV-Co (n = 6) and (**e**) LV-Cr (n = 6) resuscitation, compared with MB (n = 5). (**f**) Hierarchical clustering of Pearson correlation coefficients between gene expression of the three most significantly perturbed pathways in common with LV-Co and LV-Cr and major differentially abundant taxa.

To evaluate the relationship between the intestinal microbiota and liver (dys)function, we performed correlation analyses. Mantel testing revealed significant correlations between generalized UniFrac and AST, ALT, and total protein (Fig. 5a–c; *p* < 0.05). Likewise, there were significant correlations between Bray–Curtis and AST, ALT, and total protein (Supplementary Fig. S6). All other biomarkers were not significantly correlated (Supplementary Fig. S6 and Fig. S7). Significant correlations between α-diversity and several measured parameters were found (Supplementary Fig. S8). By far, the strongest of these correlations were found with ALT and AST suggesting a relationship between diversity and hepatocellular damage.

Given the relationship between liver injury and gut diversity (Fig. 5a–c, Supplementary Fig. S6 and Fig. S8), we hypothesized that this was mediated, in part, by perturbed liver gene expression. To test this, we sequenced reverse transcribed poly(A) transcripts from liver tissue and identified metabolic pathways altered by limited-volume resuscitation. We chose gene set analysis over individual gene analysis because small, but coordinated, changes in gene expression can have profound biological effects^26^. Forty-five and 46 pathways were perturbed by LV-Co and LV-Cr resuscitation, respectively, with 33 pathways in common (Supplementary Table S4). Among the common pathways, endoplasmic reticulum stress, intracellular ATP sensing, and oxidative phosphorylation pathways were most significantly perturbed (Fig. 5d,e). We then evaluated the correlations between these top three pathways and the major taxa that distinguished baseline (cluster 1, Fig. 4c) from dysbiosis (cluster 2, Fig. 4c). Unsupervised clustering identified three major gene clusters and two major taxonomic clusters (Fig. 5f). The taxonomic clustering grouped bacteria according to their enrichment in either baseline or dysbiosis clusters. Moreover, gene clustering revealed a pattern of inverse relationships between cluster-enriched taxa in which genes that positively correlated with cluster 1-enriched taxa were negatively correlated with cluster 2-enriched taxa, and vice versa. Collectively, these results confirm a relationship between liver (dys)function and gut dysbiosis after burn injury.

## Discussion

Fluid resuscitation is essential to the acute care of the burn patient and has improved survival and other long term outcomes. Even when surviving the initial insult, burn patients are susceptible to other complications such as hypermetabolism, sepsis, and other infectious comorbidities. Burn resuscitation has not been fine-tuned to optimize these outcomes, and initial IV fluid rates ranging from 2–4 ml-kg/%TBSA of LR per day requires many liters of fluid. To this end, we evaluated the impact of different resuscitation strategies on the gut microbiota and liver function in a swine 40% TBSA burn injury model. We found that LV-Co resuscitation exacerbated the loss of bacterial diversity and increased the risk of intestinal dysbiosis, despite achieving adequate resuscitative endpoints. Given that increasing size of burn is associated with greater levels of inflammatory cytokines and increased incidence of multiple organ dysfunction and death^27–29^, we speculate that bacterial diversity would also follow similar changes (i.e., increasing burn size may result in greater loss of alpha diversity and shift in beta diversity). Further, we showed an association between perturbed liver gene expression and the abundance of dysbiosis-associated bacteria.

Severe burns induce a state of hypovolemic shock that impairs intestinal perfusion within hours of injury^11,12,30–32^. While we found that the cross sectional area of major arteries supplying the intestine decreased to the same extent in all groups throughout the 48 h study, resuscitation with LV-Cr slightly (but significantly) reduced urine output compared to MB. In this regard, these findings suggest that LV-Co and LV-Cr resuscitation resulted in similar levels of organ perfusion, and thus, the alterations seen with LV-Co fluids are likely due to their composition. Microvascular dysfunction can cause local tissue ischemia^33^, and when combined with increased vascular permeability and decreased flow, proteins present in colloids would have increased opportunity to extravasate out of the intravascular space. The lack of exacerbated hemoconcentration in the limited-volume groups suggested that larger volumes used in MB resuscitation may not remain in the intravascular space. Fluids from MB resuscitation accumulated at sites other than injured cutaneous tissue, as there were differences in edema between injured and uninjured skin for LV-Co and LV-Cr, but not MB. Together, these results suggested that pathophysiological effects of severe burns in this model extend beyond the direct tissue damage.

Acute shifts in the gut microbiota were reported in patients with traumatic injury^15–17,34^ and severe burns^8,35^, suggesting that the gut microbiota can abruptly become dysregulated. Compared with healthy controls, the community structure of burn-injured patients was disrupted, and the bacterial composition shifted towards increased Enterobacteriaceae and reduced Bacteroidaceae and Ruminococcaceae^8^. Rodent models of burn injury have generally supported clinical observations of intestinal dysbiosis; however, reports were variable. In rats, Firmicutes and Bacteroidetes were reduced, and Proteobacteria was increased, after burn injury^20^. In contrast, Firmicutes increased after burn injury of mice^10^. At a functional level, the human microbiome is more similar to pigs than mice^36^, and pigs are also more similar to humans in regards to alimentary physiology and anatomy^37^. Additionally, the pathophysiology, hypermetabolic state, cutaneous structure, and wound healing in humans are also more similar to pigs^38^. Our results show that burn-induced dysbiosis occurs acutely (i.e., within 24 h) which was characterized by marked shifts in both α- and β-diversities, loss of Clostridia species, and enrichment of particular Gram-negative bacteria. Recent reports of intestinal dysbiosis in critically ill patients revealed that patients with extreme loss of bacterial diversity are often hypercolonized by opportunistic pathogens^39,40^ such as *Escherichia coli, Enterobacter cloacae*^41^, and *Staphylococcus aureus*^42^. We also found that several of these potentially pathogenic species (e.g., *E. coli*, *Fusobacterium* sp., *Enterococcus* sp., and *S. aureus*) were enriched in post-burn samples.

The extent in which the gut microbiome influences liver (dys)function has recently gained attention as a potential driver in chronic conditions such as inflammation^14^, but very little is known about this relationship in the acute setting of trauma. In both humans and mice, the genus *Bacteroides* was enriched in liver diseases^43,44^, and our analysis indicated that members of this genus were also enriched in post-burn pigs. Liver metabolism is central to burn pathophysiology^6^, and we found that several indicators of liver injury (i.e., aminotransferases) correlated with bacterial diversity. Since several of these enzymes are regulated post-translationally (e.g., allosteric, phosphorylation, ubiquitination), additional studies directly measuring ATP levels and enzymatic activities are necessary to confirm this.

Our data also revealed an inverse association between gene expression of major pathways that were perturbed by limited-volume resuscitation and the major differentially abundant bacteria, suggestive of a link between liver (dys)function and gut dysbiosis after burn injury. While bacterial translocation was once thought to play a role in burn-induced sepsis, this relationship highlights the possibility that bacterial products from the gut may induce stress on the liver, potentially putting the patient at risk for sepsis or multiple organ dysfunction. Upon histological examination, LV-Co tended to have more mucosal inflammation, suggesting that LV-Co may be associated with greater bacterial translocation. However, bacterial translocation was not confirmed by microbiological or molecular methods, and would need to be validated with more sensitive measures. Compared with MB, limited-volume resuscitation disrupted the transcriptional expression of several pathways in the liver, many of which involved energy metabolism and cellular stress. Surprisingly, genes involved in ATP synthesis (Complex III, IV and V), glycogen synthase, and acetyl-Co-A carboxylase trended higher with limited-volume resuscitations compared with MB, suggesting that limited-volume resuscitation may be associated with a higher cellular energetic state. It is currently not clear if these metabolic aberrations (and potentially resuscitation strategy and/or changes in the gut microbiota) influence the eventual hypermetabolic state that occurs later in burn recovery.

The impact of resuscitation on the gut microbiota are possibly influenced by both the volume and type of fluid that is administered. A previous study demonstrated that disruptions to the bacterial community after burn injury were attenuated by IV crystalloid resuscitation in a dose dependent manner^19^, suggesting that crystalloid resuscitation may benefit gut health. In the present study, LV-Co exacerbated loss of bacterial diversity and increased the risk of gut dysbiosis. To explore possible mechanisms by which LV-Co exacerbated loss of bacterial diversity, we measured biomarkers of shock, organ function, and plasma cytokines associated with inflammation. Given that both limited-volume strategies achieved comparable resuscitative and physiologic outcomes, the impact of LV-Co on the gut microbiota is in stark contrast to LV-Cr, and the mechanism by which LV-Co drives gut dysbiosis is more complex. It has been shown that perfusion influences the gut microbiome post-trauma, but our current study shows microbiome alterations in the absence of large differences in organ perfusion. It is possible that the hyper-permeable state of blood vessels due to burn injury facilitates extravasation of colloid proteins into the interstitial tissue, thereby providing growth substrates that may promote enrichment of dysbiosis-associated bacteria. To explore this possibility, we used a computational approach to predict the functional metabolic capacity of the gut microbiota after burn injury and resuscitation; however, we did not find any significant difference in metabolic pathways among resuscitation strategies. Further studies are needed to provide mechanistic insight into the effects of colloid burn resuscitation. The colloids that were chosen for this study were albumin and plasma which are natural colloids. Synthetic colloids such as hetastarch-based products are starch derivatives which could potentially have an even greater impact on bacterial metabolism and, subsequently, diversity. Our study also did not examine the effect of timing of different fluids, as natural colloids have been used 24 h post-burn when capillary leakage has slowed. Nevertheless, colloids may still have a role in reducing fluid creep and may benefit patients with fluid sensitive comorbidities, and ensuing changes in the gut microbiome may have eventual implications on outcomes such as sepsis.

There are several limitations to the current study. First, our study considers the combined effects of injury and resuscitation, and it is difficult to deconvolute these effects. Second, our model included 48 h of convalescent care and changes over a longer period of burn management was not included. Moreover, because the subject was conscious and ambulatory, a Foley catheter was not placed which precluded measurement of intra-abdominal pressure and titration of fluid levels to urine output. Since resuscitative endpoints and biomarkers of shock were not significantly different among resuscitation strategies during the ebb phase of burn injury, we speculate that limited-volume resuscitation strategies would not significantly impact survival. Finally, the standard-of-care MB resuscitation had limited number of subjects (n = 6) compared with LV-Co and LV-Cr strategies (n = 10–11), which ultimately adversely affected our statistical power.

Collectively, our findings suggest that limited-volume resuscitation did not exacerbate measured physiological derangements, but that LV-Co resuscitation adversely impacted both α- and β-bacterial diversity, and increased the risk of dysbiosis and hypercolonization by potentially pathogenic Gram-negative bacteria. Furthermore, while the gut-liver axis has been implicated in a variety of chronic disorders, we provide evidence of a linkage between liver (dys)function and the gut microbiota in the acute setting of burn injury. Whether or not these acute changes have downstream effects on burn-induced complications such as sepsis or hypermetabolism warrant further investigation.

## Methods

### Animals

This study is an extension of a previous report that examined the effect of LR alone^19^, and details on procedures and animal care can also be found there. Three-month-old female Yorkshire (*Sus scrofa*) pigs (n = 30, 41.0 ± 3.1 kg) were included in this report. Animals had a minimum seven-day acclimation period where they were singly housed, with ad libitum access to water, and fed a maintenance commercial laboratory porcine formulated pelleted diet (Laboratory Mini-Pig Grower Diet, Cat # 5081, LabDiet, Richmond, IN). Animals were randomly allocated upon delivery such that each of the following five treatment groups were represented evenly in each litter: limited volume fluids (i.e. 15 mL/kg/day) in the form of LR (n = 6), PL (n = 6), AB (n = 6), or blood-type matched porcine unpooled FFP (n = 6). The MB formula prescribed at 80 mL/kg/day of LR served as the standard-of-care resuscitation (n = 6).

### Burn injury and post-injury care

Prior to the burn injury, analgesia was achieved with intramuscular Buprenex-HCl Sustained Release (0.1–0.24 mg/kg, Veterinary Technologies/ZooPharm, Windsor, CO). The 40% TBSA burn was performed via contact burns as previously described^38^. Briefly, animals were sedated with intramuscular Telazol (6 mg/kg), intubated, and ventilated (initial tidal volume 10 mL/kg, peak inspiratory pressure 20 cmH_2_O) with respiratory adjustment to achieve an end-tidal PCO_2_ of 40 ± 5 mmHg, and anesthesia with isoflurane (1–3%). Hair was removed with clippers and razors, and external jugular vein catheters were placed, which exited the nape of the neck. Large (9 × 15 cm) and small (5 × 5 cm) custom-designed brass blocks were heated 100 ± 0.2 ºC and placed against the skin for 30 s for full-thickness burns^45^. Wounds were covered with Ioban antimicrobial dressings (3M, St. Paul, MN) for the duration of the experiment.

### Burn wounds and post-injury follow up

After injury, animals were kept in metabolic cages for control of fluid input/output in a conscious fashion^46^, and were allowed access to standard feed once standing independently. Animals distressed with the new environment were administered intramuscular midazolam (0.1–0.25 mg/kg). Approximately 24 h and 48 h post-burn, animals were lightly sedated with Telazol (6 mg/kg) to collect blood samples and rectal swabs. Once anesthetized, a rectal swab was taken using a sterile, individually wrapped swab that was inserted ca. 3 in. into the rectum. Swabs were immediately stored at − 80 °C until processed. Blood gas analytes (lactate, pH) were immediately measured with iSTAT CG4 + test cartridges (Abbott, Abbott Park, IL), while all other biochemical and blood cell analytes were analyzed on Siemens Dimension Xp Plus, and Abbott Cell-Dyn 3700 systems, respectively. At 48 h post-injury, animals were humanely euthanized and ~ 15 g of burned and unburned skin was excised, weighed, and dried at 58 °C for seven days for wet-to-dry weight.

### Contrast-enhanced computed tomography (CT)

While under anesthesia at baseline and 48 h post-injury, 40 ml Iopamidol (755 mg/ml) was injected IV for CT-angiographies. Scans were acquired on an Aquilion Prime 160-slice CT scanner (Toshiba America Medical Systems, Tustin, CA)**,** and analyzed with Vitrea Software (Vital Images Inc., Minnetonka, MN) using the Vascular: Runoff protocol. After bones and soft tissues were digitally subtracted, superior and inferior mesenteric arteries (SMA and IMA, respectively) were selected and cross-sectional areas measured using the lumen and wall vessel tools. The SMA was taken 0.5–1.0 mm from the first branch point, while the IMA was taken 10 mm from the aorta, and only images of arteries with sufficient contrast were included in analysis.

### Plasma cytokines

Blood was collected at the indicated time-points in K_2_EDTA-treated Vacutainer tubes (Becton Dickinson and Company, Franklin Lakes, NJ) and plasma was harvested and stored at − 80 °C. A panel of nine cytokines (GM-CSF, IFNγ, IL-1β, IL-1ra, IL-4, IL-6, IL-8, IL-10, IL-18) was quantified using the MILLIPLEX MAP Porcine Cytokine/Chemokine (EMD Millipore, Burlington MA) kit according to the manufacturer’s recommendations. Analytes that were below the limit of quantification for ≥ 50% of the samples were excluded from further analyses. Values for censored samples were substituted with the limit of detection/$\sqrt{2}$ ^47^.

### Histology

At 48 h post-burn, portions of the duodenum were fixed in formalin. The fixed specimens were embedded in paraffin, sectioned, and stained using a modified Gran stain procedure^48^. Images were acquired on an Axio Scan.Z1 slide scanner (Zeiss Microscopy, White Plains NY) and analyzed with the ZEN2 software. A researcher blinded to the sample treatment scored the tissue sections on an ordinal scale of 0–4 for changes in villi architecture and mucosal inflammation (0 = none, 1 = minimal, 2 = mild, 3 = moderate, 4 = marked).

### 16S rRNA gene sequencing and data processing

DNA was extracted from rectal swabs using the QIAmp PowerFecal DNA Kit (Qiagen, Germantown, MD) according to the manufacturer’s recommendations with the following modification: swab tips were aseptically removed, placed into the dry bead tube, and wrung against the side of the tube after the bead beating step. DNA quality and quantity were assessed by NanoDrop and Qubit (ThermoFisher Scientific, Waltham, MA). The V1/V2 regions of 16S rRNA gene were amplified using primers 27F and 355R^49^. Sequencing libraries were constructed using the Nextera XT Index Kit v2 and sequenced on the Illumina MiSeq platform using the MiSeq Reagent Kit v3 600-cycle (Illumina San Diego, CA).

Demultiplexed FASTQ files were processed using QIIME2 v2019.7^50^. The DADA2 pipeline for paired-end reads was used to remove primers, filter chimeras, and quality control sequencing reads^51^. Reads were truncated at the first instance of a base quality of ≤ 2 or median quality score of < 30. Amplified sequence variants were aligned using Multiple Alignment using Fast Fourier Transform and representative sequence variants were used to construct a phylogenetic tree using FastTree. Taxonomic classification was based on the GreenGenes 13_8 99% OTU database using a Naïve Bayes classifier. For differential abundance analysis, OTU tables were first filtered to remove singletons and doubletons. For library normalization, each sample was rarefied to the lowest library sequencing depth (50,000 reads) with q2-rarefy, which retained 100% of samples. PICRUSt2 v2.2.0b was used to predict functional metagenomics using 16S rRNA reads^52^. Differential abundance of MetaCyc pathways were evaluated by ALDEx2 using the glm algorithm which incorporated the Benjamini–Hochberg correction procedure^53^.

### Nanopore poly(A) RNA sequencing and data processing

RNA was extracted from a subset of liver samples from FFP (n = 3), AB (n = 3), LR (n = 3), PL (n = 3), and MB (n = 5) subjects using the RNAzol RT (Molecular Research Center, Cincinnati, OH) reagent according to the manufacturer’s recommendations with an additional chloroform extraction step. Briefly, the resuspended RNA pellet was combined with an equal volume of chloroform, mixed by gentle vortexing, and centrifuged. The aqueous phase was combined with 1/10th volume of 3M sodium acetate (pH 5.5) and 0.8 × volume of isopropanol. The mixture was incubated at − 80 °C for 1 h and then centrifuged to pellet the RNA. RNA quality and quantity were evaluated by NanoDrop, Qubit, and BioAnalyzer assays. Total RNA was reverse transcribed and barcoded according to the Oxford Nanopore’s PCR-cDNA Barcoding recommendations (Oxford Nanopore Technologies, UK). The library was sequenced on MinION Mk1C with FLO-MIN106D R9 flow cells (Oxford Nanopore Technologies). Data were basecalled and demultiplexed with Guppy v3.2.4. Reads were mapped to the ensemble *Sus scrofa* 11.1.96 reference transcriptome with Minimap2 v2.16-r922^54^ and the output was converted to BAM format using SAMtools v1.9^55^. Salmon v0.13.1^56^ was used to count cDNA sequencing reads.

### Statistical analyses

All data were natural-log transformed when necessary and analyzed in either GraphPad Prism (v8.1.2) or R (v3.5.1). Clinical biomarkers and bacterial diversities were first analyzed for differences between FFP and AB (colloids), and between PL and LR (crystalloids). Total bilirubin and urine output differed significantly between colloid treatments. No significant differences were found for other clinical biomarkers and measures of diversity (Supplementary Fig. S9); thus, FFP and AB groups were collapsed to LV-Co resuscitation, and PL and LR groups were collapsed to LV-Cr resuscitation. Clinical biomarker data were centered and scaled prior to computing principal components (prcomp), and visualizing PCA biplots (factoextra v1.0.6). Urine output, edema, and CT data were analyzed by linear mixed effects (LME) models or two-way ANOVA. Plasma cytokines and histological scores were analyzed by Wilcoxon rank-sum tests. All statistical tests incorporated the Holm multiple comparisons correction at α = 0.05.

Differential bacterial abundance testing was performed using Linear Discriminant Analysis Effect Size (LEfSe) with the one-against-all parameter for the multi-class analysis ^57^. Hierarchical clustering was performed with Bray–Curtis and visualized by heatmap (gplots v3.0.3). Bootstrapping was conducted with a resampling size of 100. Alpha and β-diversity indices were calculated on rarefied counts (q2-diversity). Generalized UniFrac distances were calculated with α = 0.5^58^. Longitudinal analyses was performed by extracting first distances (q2-longitudinal)^25^ with pre-injury as baseline. Separate LME models (nlme v3.1)^59^, adjusting for midazolam use, were fitted to examine each α- and β-diversity metric. Mantel tests with 999 permutations (ade4 v1.7) evaluated distance correlations.

For Nanopore poly(A) RNA sequence analyses, gene counts were first normalized by the TMM method (edgeR v3.24.3)^60,61^. Then, enriched pathways were identified based on the KEGG *Sus scrofa* signaling and metabolism gene sets evaluating two-direction changes simultaneously (GAGE v2.32.1)^26^. All pathway analyses used MB as the reference condition to which LV-Co and LV-Cr were compared. Adjustments for false discovery rate were described previously^62^. Correlations between gene expression (CPM transformed) and bacterial abundance (rarefied natural log-transformed counts) were tested with Pearson’s correlation.

### Ethics approval

Research was conducted in compliance with the Animal Welfare Act, the implementing Animal Welfare Regulations, and the principles of the Guide for the Care and Use of Laboratory Animals, National Research Council. The facility's Institutional Animal Care and Use Committee approved all research conducted in this study. The facility where this research was conducted is fully accredited by AAALAC.

### Consent for publication

All authors have approved the manuscript for submission.

### Disclaimer

The views expressed in this article are those of the author(s) and do not reflect the official policy or position of the U.S. Army Medical Department, Uniformed Services University, Department of the Army, DoD, or the U.S. Government.

## Supplementary information

Supplementary Information.

## Data Availability

The 16S rRNA gene sequencing dataset generated and analyzed during the current study are available in the SRA repository, accession PRJNA629013. Due to the size of raw HDF5 transcriptome files, datasets are available upon request.
