# Supplementary material for: Burn resuscitation strategy influences the gut microbiota-liver axis in swine
Source: Sci Rep. 2020 Sep 24;10:15655. doi: 10.1038/s41598-020-72511-8 (PMC7515893; doi:10.1038/s41598-020-72511-8)
Supplement: Supplementary file 1 — Supplementary Information. [file 41598_2020_72511_MOESM1_ESM.pdf]

Supplementary Information

Burn resuscitation strategy influences the gut microbiota-liver axis in swine

Wayne T. Muraoka[1], Jose C. Granados[1], Belinda I. Gomez[1], Susannah E. Nicholson[2], Kevin K. Chung[1,3], Jeffrey W. Shupp[4], James A. Bynum[1], Michael A. Dubick[1], David M. Burmeister[1,3,*]

[1] Blood Coagulation Research Department, United States Army Institute of Surgical Research, JBSA Ft. Sam Houston, TX 78234

[2] Division of Trauma and Emergency Surgery, Department of Surgery, University of Texas Health Science Center, San Antonio TX 78229

[3] Department of Medicine, Uniformed Services University of the Health Sciences, Bethesda, MD, 20814

[4] The Burn Center, MedStar Washington Hospital Center, Department of Surgery, Georgetown University School of Medicine, Washington, DC 20010

W.T.M (wayne.muraoka.ctr@mail.mil), J.C.G. (jose.c.granados.ctr@mail.mil), B.I.G. (bgomez167@alamo.edu), S.E.N. (nicholsons@uthscsa.edu), K.K.C. (kevin.chung@usuhs.edu), J.W.S. (Jeffrey.W.Shupp@medstar.net) , J.A.B. (james.bynum.civ@mail.mil), M.A.D. (Michael.a.dubick.civ@mail.mil), D.M.B (david.burmeister@usuhs.mil)

Included in this file are:

Table S1. Blood gas and biochemistry values by post-injury time and resuscitation strategy.

Table S2. Differential abundance over time identified by LEfSe.

Table S3. Differential abundance across resuscitation identified by LEfSe.

Table S4. Pathways perturbed by limited volume resuscitation.

Figure S1. Additional analyses of limited-volume resuscitation impact on burn pathophysiology.

Figure S2. Changes in plasma cytokines after burn and resuscitation.

Figure S3. Villus architecture and inflammation after burn and resuscitation.

Figure S4. Changes in bacterial composition after burn and resuscitation.

Figure S5. Within resuscitation-group dispersion of Bray-Curtis and generalized UniFrac using spatial medians.

Figure S6. Correlation analyses of clinical biomarkers and Bray-Curtis.

Figure S7. Clinical biomarkers not significantly correlated with generalized UniFrac.

Figure S8. Associations of alpha diversities and clinical biomarkers.

Figure S9. Bacterial diversity between AB and FFP, and LR and PL.

Table S1. Blood gas and biochemistry values by post-injury time and resuscitation strategy. Values indicate mean and SD. ALT: alanine aminotransferase, AST: aspartate aminotransferase, BUN: blood urea nitrogen, CKI: creatine kinase, Creat: creatinine, GLUC: glucose, HCT: hematocrit, HGB: hemoglobin, LAC: lactate, PLT: platelet count, RBC: red blood cell count, TBI: total bilirubin, TP: total protein, WBC: white blood cell count. **: Tukey's $p < 0.01$ for LV-Co vs MB.

	LV-Co			LV-Cr			MB		
	BL (n = 10)	24 h (n = 10)	48 h (n = 9)	BL (n = 11)	24 h (n = 11)	48 h (n = 11)	BL (n = 5)	24 h (n = 6)	48 h (n = 6)
ALT	70.3 ± 15.97	107.4 ± 16.26	107.78 ± 15.74	70.45 ± 13.25	114.27 ± 12.34	113.36 ± 12.72	60.2 ± 14.02	102.83 ± 19.14	99 ± 18.6
AST	33.1 ± 9.73	78.2 ± 11.09	65 ± 10.17	29.45 ± 4.76	62 ± 11.7	55.91 ± 16.32	29.4 ± 2.7	79 ± 20.62	62.33 ± 11.78
BUN	9.72 ± 1.65	17.53 ± 2.5	14.46 ± 2.72	10.05 ± 2.63	19.95 ± 6.03	15.25 ± 4.69	8.18 ± 2.3	14.55 ± 2.84	9.7 ± 1.86
CKI	530 ± 166	5916 ± 3313	1970 ± 1034	599 ± 314	3959 ± 2414	1625 ± 926	647 ± 371	3107 ± 1666	1319 ± 613
Creat	1.41 ± 0.15	1.56 ± 0.17	1.44 ± 0.14	1.37 ± 0.19	1.67 ± 0.27	1.45 ± 0.19	1.23 ± 0.26	1.39 ± 0.12	1.17 ± 0.11
GLUC	63.9 ± 8.28	193.6 ± 71.78	132.78 ± 38.83	58.45 ± 19.66	195.27 ± 74.94	206.73 ± 114.55	79 ± 13.23	159.5 ± 41.07	130.67 ± 54.71
HCT	27.91 ± 2.59	33.45 ± 1.88	31.32 ± 3.43	28.18 ± 2.13	36.3 ± 4.86	31.43 ± 3.28	31.28 ± 3.47	33.05 ± 4.72	28.2 ± 2.69
HGB	9.1 ± 0.81	10.84 ± 0.61	10.11 ± 0.95	9.12 ± 0.81	11.73 ± 1.65	10.07 ± 1.22	10.08 ± 0.87	10.53 ± 1.3	9.2 ± 0.77
LAC	1.8 ± 0.5	1.2 ± 0.29	1.44 ± 1.26	1.43 ± 0.45	1.82 ± 1.59	1.16 ± 0.7	1.56 ± 0.3	1.28 ± 0.55	0.81 ± 0.28
pH	7.43 ± 0.03	7.40 ± 0.02	7.43 ± 0.02	7.44 ± 0.04	7.36 ± 0.04	7.41 ± 0.02	7.42 ± 0.02	7.4 ± 0.03	7.43 ± 0.03
PLT	315.1 ± 74.47	278 ± 61.41	257.89 ± 50.63	353.64 ± 76.55	333.36 ± 58.69	288.73 ± 67.87	267.2 ± 46.53	272.33 ± 81.95	238.33 ± 72.54
RBC	5.17 ± 0.49	6.16 ± 0.55	5.76 ± 0.69	5.43 ± 0.46	6.93 ± 0.85	6.03 ± 0.66	5.76 ± 0.37	6.06 ± 0.54	5.26 ± 0.29
TBI	0.08 ± 0.05	0.18 ± 0.12	0.31 ± 0.3	0.1 ± 0.05	0.15 ± 0.08	0.16 ± 0.11	0.1 ± 0.06	0.11 ± 0.07	0.2 ± 0.14
TP**	5.51 ± 0.32	6.53 ± 0.25	6.86 ± 0.31	5.38 ± 0.54	6.35 ± 0.86	6.23 ± 0.67	5.3 ± 0.19	5.55 ± 0.54	5.33 ± 0.41
WBC	17.07 ± 2.6	19.93 ± 3.35	19.12 ± 4.22	15.84 ± 2.53	20.39 ± 5.49	18.05 ± 4.93	17.83 ± 4.51	18.56 ± 3.71	18.57 ± 4.61

Table S2. Differential abundance over time identified by LEfSe. Bold taxa are most abundant (by sequence count) and visualized in Figure 4c. Differentially abundant clades were defined as *p* < 0.05 and LDA > 2.

Clade	Time	Log_{10} LDA	*p*-value
k__Bacteria.p__Actinobacteria.c__Actinobacteria.o__Actinomycetales.f__Micrococcaceae.g__	48	2.778447	0.001984
k__Bacteria.p__Actinobacteria.c__Actinobacteria.o__Actinomycetales.f__Micrococcaceae.g__Kocuria	0	3.494125	0.014577
k__Bacteria.p__Actinobacteria.c__Coriobacteriia.o__Coriobacteriales.f__Coriobacteriaceae.__	0	2.756682	0.000892
k__Bacteria.p__Actinobacteria.c__Coriobacteriia.o__Coriobacteriales.f__Coriobacteriaceae.g__	0	2.664545	1.02E-06
k__Bacteria.p__Actinobacteria.c__Coriobacteriia.o__Coriobacteriales.f__Coriobacteriaceae.g__Adlercreutzia	0	3.130184	1.88E-05
k__Bacteria.p__Bacteroidetes	0	4.786285	0.001404
k__Bacteria.p__Bacteroidetes.c__Bacteroidia	0	4.786239	0.001404
k__Bacteria.p__Bacteroidetes.c__Bacteroidia.o__Bacteroidales	0	4.786239	0.001404
k__Bacteria.p__Bacteroidetes.c__Bacteroidia.o__Bacteroidales.__	0	3.85344	7.73E-07
k__Bacteria.p__Bacteroidetes.c__Bacteroidia.o__Bacteroidales.__.__	0	3.85344	7.73E-07
k__Bacteria.p__Bacteroidetes.c__Bacteroidia.o__Bacteroidales.f___Paraprevotellaceae_.__	0	3.085687	2.56E-06
k__Bacteria.p__Bacteroidetes.c__Bacteroidia.o__Bacteroidales.f___Paraprevotellaceae_.g__	0	2.951864	5.78E-05
k__Bacteria.p__Bacteroidetes.c__Bacteroidia.o__Bacteroidales.f___Paraprevotellaceae_.g__CF231	0	3.311981	2.51E-09
k__Bacteria.p__Bacteroidetes.c__Bacteroidia.o__Bacteroidales.f___Paraprevotellaceae_.g__YRC22	0	3.326432	6.02E-07
k__Bacteria.p__Bacteroidetes.c__Bacteroidia.o__Bacteroidales.f__Bacteroidaceae	48	4.873383	3.29E-07
**k__Bacteria.p__Bacteroidetes.c__Bacteroidia.o__Bacteroidales.f__Bacteroidaceae.g__Bacteroides**	48	4.873383	3.29E-07
k__Bacteria.p__Bacteroidetes.c__Bacteroidia.o__Bacteroidales.f__Prevotellaceae	0	4.832724	6.02E-08
k__Bacteria.p__Bacteroidetes.c__Bacteroidia.o__Bacteroidales.f__Prevotellaceae.__	24	3.155261	0.032317
**k__Bacteria.p__Bacteroidetes.c__Bacteroidia.o__Bacteroidales.f__Prevotellaceae.g__**	0	3.549463	1.72E-05
**k__Bacteria.p__Bacteroidetes.c__Bacteroidia.o__Bacteroidales.f__Prevotellaceae.g__Prevotella**	0	4.81547	4.11E-08
k__Bacteria.p__Bacteroidetes.c__Bacteroidia.o__Bacteroidales.f__RF16	0	3.853411	4.35E-09
**k__Bacteria.p__Bacteroidetes.c__Bacteroidia.o__Bacteroidales.f__RF16.g__**	0	3.853411	4.35E-09
k__Bacteria.p__Bacteroidetes.c__Bacteroidia.o__Bacteroidales.f__Rikenellaceae	0	2.543845	0.024852
k__Bacteria.p__Bacteroidetes.c__Bacteroidia.o__Bacteroidales.f__Rikenellaceae.g__	0	2.536339	0.024852
k__Bacteria.p__Bacteroidetes.c__Bacteroidia.o__Bacteroidales.f__S24_7	0	3.817694	0.000411
**k__Bacteria.p__Bacteroidetes.c__Bacteroidia.o__Bacteroidales.f__S24_7.g__**	0	3.817694	0.000411
k__Bacteria.p__Cyanobacteria	0	3.559007	8.85E-12
k__Bacteria.p__Cyanobacteria.c__4C0d_2	0	3.559007	8.85E-12

k__Bacteria.p__Cyanobacteria.c__4C0d_2.o__YS2	0	3.559007	8.85E-12
k__Bacteria.p__Cyanobacteria.c__4C0d_2.o__YS2.f__	0	3.559007	8.85E-12
k__Bacteria.p__Cyanobacteria.c__4C0d_2.o__YS2.f__.g__	0	3.559007	8.85E-12
k__Bacteria.p__Elusimicrobia	0	3.265216	2.53E-06
k__Bacteria.p__Elusimicrobia.c__Elusimicrobia	0	3.265216	2.53E-06
k__Bacteria.p__Elusimicrobia.c__Elusimicrobia.o__Elusimicrobiales	0	3.265216	2.53E-06
k__Bacteria.p__Elusimicrobia.c__Elusimicrobia.o__Elusimicrobiales.f__Elusimicrobiaceae	0	3.265216	2.53E-06
k__Bacteria.p__Elusimicrobia.c__Elusimicrobia.o__Elusimicrobiales.f__Elusimicrobiaceae.g__	0	3.265216	2.53E-06
k__Bacteria.p__Fibrobacteres	0	3.053709	0.00178
k__Bacteria.p__Fibrobacteres.c__Fibrobacteria	0	3.053709	0.00178
k__Bacteria.p__Fibrobacteres.c__Fibrobacteria.o__Fibrobacterales	0	3.053709	0.00178
k__Bacteria.p__Fibrobacteres.c__Fibrobacteria.o__Fibrobacterales.f__Fibrobacteraceae	0	3.053709	0.00178
k__Bacteria.p__Fibrobacteres.c__Fibrobacteria.o__Fibrobacterales.f__Fibrobacteraceae.g__Fibrobacter	0	3.053709	0.00178
k__Bacteria.p__Firmicutes	0	4.85178	4.26E-06
k__Bacteria.p__Firmicutes.c__Bacilli.o__Bacillales	48	3.465616	0.000183
k__Bacteria.p__Firmicutes.c__Bacilli.o__Bacillales.f__Staphylococcaceae	48	3.459209	8.8E-05
k__Bacteria.p__Firmicutes.c__Bacilli.o__Bacillales.f__Staphylococcaceae.g__Staphylococcus	48	3.459014	8.8E-05
k__Bacteria.p__Firmicutes.c__Bacilli.o__Lactobacillales.f__Enterococcaceae	48	3.486311	0.000888
k__Bacteria.p__Firmicutes.c__Bacilli.o__Lactobacillales.f__Enterococcaceae.g__Enterococcus	48	3.486409	0.000888
k__Bacteria.p__Firmicutes.c__Bacilli.o__Lactobacillales.f__Lactobacillaceae	0	4.042648	0.000998
**k__Bacteria.p__Firmicutes.c__Bacilli.o__Lactobacillales.f__Lactobacillaceae.g__Lactobacillus**	0	4.042648	0.000998
k__Bacteria.p__Firmicutes.c__Clostridia	0	4.797335	2.19E-06
k__Bacteria.p__Firmicutes.c__Clostridia.o__Clostridiales	0	4.797335	2.19E-06
k__Bacteria.p__Firmicutes.c__Clostridia.o__Clostridiales.__	0	3.996386	6.45E-08
k__Bacteria.p__Firmicutes.c__Clostridia.o__Clostridiales.__.__	0	3.996386	6.45E-08
k__Bacteria.p__Firmicutes.c__Clostridia.o__Clostridiales.f___Mogibacteriaceae_	0	3.232029	1.64E-07
k__Bacteria.p__Firmicutes.c__Clostridia.o__Clostridiales.f___Mogibacteriaceae_.g__	0	3.218434	2.08E-07
k__Bacteria.p__Firmicutes.c__Clostridia.o__Clostridiales.f___Mogibacteriaceae_.g__Mogibacterium	0	2.813414	0.000934
k__Bacteria.p__Firmicutes.c__Clostridia.o__Clostridiales.f__Clostridiaceae	0	3.028942	0.000215
k__Bacteria.p__Firmicutes.c__Clostridia.o__Clostridiales.f__Clostridiaceae.g__Clostridium	0	2.931462	2.16E-05
k__Bacteria.p__Firmicutes.c__Clostridia.o__Clostridiales.f__Dehalobacteriaceae	0	2.946592	0.000689
k__Bacteria.p__Firmicutes.c__Clostridia.o__Clostridiales.f__Dehalobacteriaceae.g__Dehalobacterium	0	2.845808	0.000392
k__Bacteria.p__Firmicutes.c__Clostridia.o__Clostridiales.f__Lachnospiraceae	0	4.3835	6.27E-10
k__Bacteria.p__Firmicutes.c__Clostridia.o__Clostridiales.f__Lachnospiraceae.__	0	3.969879	1.75E-10
**k__Bacteria.p__Firmicutes.c__Clostridia.o__Clostridiales.f__Lachnospiraceae.g__**	0	3.531664	4.48E-09
k__Bacteria.p__Firmicutes.c__Clostridia.o__Clostridiales.f__Lachnospiraceae.g__Anaerostipes	0	2.938615	3.57E-08

k__Bacteria.p__Firmicutes.c__Clostridia.o__Clostridiales.f__Lachnospiraceae.g__Blautia	0	3.019793	0.018303
k__Bacteria.p__Firmicutes.c__Clostridia.o__Clostridiales.f__Lachnospiraceae.g__Clostridium	0	2.472933	0.01066
k__Bacteria.p__Firmicutes.c__Clostridia.o__Clostridiales.f__Lachnospiraceae.g__Coprococcus	0	3.506917	5.4E-10
k__Bacteria.p__Firmicutes.c__Clostridia.o__Clostridiales.f__Lachnospiraceae.g__Dorea	0	3.048915	1.61E-05
k__Bacteria.p__Firmicutes.c__Clostridia.o__Clostridiales.f__Lachnospiraceae.g__Lachnobacterium	0	2.612802	4.67E-07
k__Bacteria.p__Firmicutes.c__Clostridia.o__Clostridiales.f__Lachnospiraceae.g__Lachnospira	0	3.584943	6.07E-09
k__Bacteria.p__Firmicutes.c__Clostridia.o__Clostridiales.f__Lachnospiraceae.g__Roseburia	0	3.023242	9.17E-08
k__Bacteria.p__Firmicutes.c__Clostridia.o__Clostridiales.f__Lachnospiraceae.g__Shuttleworthia	0	2.970222	3.83E-07
k__Bacteria.p__Firmicutes.c__Clostridia.o__Clostridiales.f__Peptococcaceae	0	2.564686	0.001654
k__Bacteria.p__Firmicutes.c__Clostridia.o__Clostridiales.f__Peptococcaceae.g__rc4_4	0	2.576458	1.52E-06
k__Bacteria.p__Firmicutes.c__Clostridia.o__Clostridiales.f__Peptostreptococcaceae	48	2.420837	0.041107
k__Bacteria.p__Firmicutes.c__Clostridia.o__Clostridiales.f__Peptostreptococcaceae.g__	0	2.656965	4.26E-05
k__Bacteria.p__Firmicutes.c__Clostridia.o__Clostridiales.f__Ruminococcaceae	0	4.565533	1.33E-07
k__Bacteria.p__Firmicutes.c__Clostridia.o__Clostridiales.f__Ruminococcaceae.__	0	3.525542	4.25E-05
**k__Bacteria.p__Firmicutes.c__Clostridia.o__Clostridiales.f__Ruminococcaceae.g__**	0	4.187435	9.71E-07
k__Bacteria.p__Firmicutes.c__Clostridia.o__Clostridiales.f__Ruminococcaceae.g__Faecalibacterium	0	3.361438	8.91E-06
**k__Bacteria.p__Firmicutes.c__Clostridia.o__Clostridiales.f__Ruminococcaceae.g__Oscillospira**	0	3.919182	3.59E-07
**k__Bacteria.p__Firmicutes.c__Clostridia.o__Clostridiales.f__Ruminococcaceae.g__Ruminococcus**	0	3.876563	1.78E-09
k__Bacteria.p__Firmicutes.c__Clostridia.o__Clostridiales.f__Veillonellaceae.g__	0	2.84918	5E-05
k__Bacteria.p__Firmicutes.c__Clostridia.o__Clostridiales.f__Veillonellaceae.g__Anaerovibrio	0	3.056162	2.22E-09
k__Bacteria.p__Firmicutes.c__Clostridia.o__Clostridiales.f__Veillonellaceae.g__Mitsuokella	0	2.803711	0.01638
k__Bacteria.p__Firmicutes.c__Clostridia.o__Clostridiales.f__Veillonellaceae.g__Phascolarctobacterium	0	3.361728	3.14E-07
**k__Bacteria.p__Firmicutes.c__Clostridia.o__Clostridiales.f__Veillonellaceae.g__Veillonella**	48	3.72175	1.31E-06
k__Bacteria.p__Firmicutes.c__Erysipelotrichi	0	3.62112	0.011421
k__Bacteria.p__Firmicutes.c__Erysipelotrichi.o__Erysipelotrichales	0	3.62112	0.011421
k__Bacteria.p__Firmicutes.c__Erysipelotrichi.o__Erysipelotrichales.f__Erysipelotrichaceae	0	3.62112	0.011421
k__Bacteria.p__Firmicutes.c__Erysipelotrichi.o__Erysipelotrichales.f__Erysipelotrichaceae.__	0	2.892681	1.79E-05
k__Bacteria.p__Firmicutes.c__Erysipelotrichi.o__Erysipelotrichales.f__Erysipelotrichaceae.g__	0	2.738721	4.3E-06
k__Bacteria.p__Firmicutes.c__Erysipelotrichi.o__Erysipelotrichales.f__Erysipelotrichaceae.g__Asteroleplasma	0	2.544699	0.022003
k__Bacteria.p__Firmicutes.c__Erysipelotrichi.o__Erysipelotrichales.f__Erysipelotrichaceae.g__Bulleidia	0	3.052822	0.000204
k__Bacteria.p__Firmicutes.c__Erysipelotrichi.o__Erysipelotrichales.f__Erysipelotrichaceae.g__Coprobacillus	0	2.614913	0.000127
k__Bacteria.p__Firmicutes.c__Erysipelotrichi.o__Erysipelotrichales.f__Erysipelotrichaceae.g__L7A_E11	0	2.590308	0.000146
k__Bacteria.p__Firmicutes.c__Erysipelotrichi.o__Erysipelotrichales.f__Erysipelotrichaceae.g__p_75_a5	0	3.209656	0.000607
k__Bacteria.p__Firmicutes.c__Erysipelotrichi.o__Erysipelotrichales.f__Erysipelotrichaceae.g__RFN20	0	2.912698	0.000477
k__Bacteria.p__Fusobacteria	48	4.078204	0.000471
k__Bacteria.p__Fusobacteria.c__Fusobacteriia	48	4.081449	0.000471

k__Bacteria.p__Fusobacteria.c__Fusobacteriia.o__Fusobacteriales	48	4.075177	0.000471
k__Bacteria.p__Fusobacteria.c__Fusobacteriia.o__Fusobacteriales.f__Fusobacteriaceae	48	4.079252	0.000471
**k__Bacteria.p__Fusobacteria.c__Fusobacteriia.o__Fusobacteriales.f__Fusobacteriaceae. g__Fusobacterium**	48	4.076634	0.000471
k__Bacteria.p__Lentisphaerae	0	2.336118	0.000117
k__Bacteria.p__Lentisphaerae.c___Lentisphaeria_	0	2.338706	0.000117
k__Bacteria.p__Lentisphaerae.c___Lentisphaeria_.o__Victivallales	0	2.475903	0.002019
k__Bacteria.p__Lentisphaerae.c___Lentisphaeria_.o__Victivallales.f__Victivallaceae	0	2.482957	0.002019
k__Bacteria.p__Lentisphaerae.c___Lentisphaeria_.o__Victivallales.f__Victivallaceae.g__	0	2.698911	0.005519
k__Bacteria.p__Lentisphaerae.c___Lentisphaeria_.o__Z20	0	2.335434	0.00034
k__Bacteria.p__Lentisphaerae.c___Lentisphaeria_.o__Z20.f__R4_45B	0	2.335135	0.00034
k__Bacteria.p__Lentisphaerae.c___Lentisphaeria_.o__Z20.f__R4_45B.g__	0	2.334409	0.00034
k__Bacteria.p__Proteobacteria	24	5.122348	1.96E-06
k__Bacteria.p__Proteobacteria.c__Alphaproteobacteria	0	3.183585	5.4E-10
k__Bacteria.p__Proteobacteria.c__Alphaproteobacteria.__	0	2.257383	0.008097
k__Bacteria.p__Proteobacteria.c__Alphaproteobacteria.__.__	0	2.249512	0.008097
k__Bacteria.p__Proteobacteria.c__Alphaproteobacteria.__.__.__	0	2.271704	0.008097
k__Bacteria.p__Proteobacteria.c__Alphaproteobacteria.o__	0	2.578165	0.004034
k__Bacteria.p__Proteobacteria.c__Alphaproteobacteria.o__.f__	0	2.578043	0.004034
k__Bacteria.p__Proteobacteria.c__Alphaproteobacteria.o__.f__.g__	0	2.578244	0.004034
k__Bacteria.p__Proteobacteria.c__Alphaproteobacteria.o__RF32	0	2.814036	1.22E-09
k__Bacteria.p__Proteobacteria.c__Alphaproteobacteria.o__RF32.f__	0	2.814014	1.22E-09
k__Bacteria.p__Proteobacteria.c__Alphaproteobacteria.o__RF32.f__.g__	0	2.813845	1.22E-09
k__Bacteria.p__Proteobacteria.c__Alphaproteobacteria.o__Rickettsiales	0	2.691614	1.43E-09
k__Bacteria.p__Proteobacteria.c__Alphaproteobacteria.o__Rickettsiales.f__	0	2.689706	1.43E-09
k__Bacteria.p__Proteobacteria.c__Alphaproteobacteria.o__Rickettsiales.f__.g__	0	2.694252	1.43E-09
k__Bacteria.p__Proteobacteria.c__Betaproteobacteria	0	3.510172	3.71E-08
k__Bacteria.p__Proteobacteria.c__Betaproteobacteria.__	0	2.265848	7.42E-05
k__Bacteria.p__Proteobacteria.c__Betaproteobacteria.__.__	0	2.267229	7.42E-05
k__Bacteria.p__Proteobacteria.c__Betaproteobacteria.__.__.__	0	2.235738	7.42E-05
k__Bacteria.p__Proteobacteria.c__Betaproteobacteria.o__Burkholderiales	0	3.332829	3.24E-08
k__Bacteria.p__Proteobacteria.c__Betaproteobacteria.o__Burkholderiales.f__Alcaligenaceae	0	3.248997	2.18E-07
k__Bacteria.p__Proteobacteria.c__Betaproteobacteria.o__Burkholderiales.f__Alcaligenaceae.g__Sutterella	0	3.248997	2.18E-07
k__Bacteria.p__Proteobacteria.c__Betaproteobacteria.o__Burkholderiales.f__Oxalobacteraceae	0	2.588174	0.00123
k__Bacteria.p__Proteobacteria.c__Betaproteobacteria.o__Burkholderiales.f__Oxalobacteraceae. g__Oxalobacter	0	2.588174	0.00123
k__Bacteria.p__Proteobacteria.c__Betaproteobacteria.o__Tremblayales	0	2.553735	1.59E-05

k__Bacteria.p__Proteobacteria.c__Betaproteobacteria.o__Tremblayales.f__	0	2.555325	1.59E-05
k__Bacteria.p__Proteobacteria.c__Betaproteobacteria.o__Tremblayales.f__.g__	0	2.553516	1.59E-05
k__Bacteria.p__Proteobacteria.c__Epsilonproteobacteria	24	4.341769	0.001587
k__Bacteria.p__Proteobacteria.c__Epsilonproteobacteria.o__Campylobacterales	24	4.341769	0.001587
**k__Bacteria.p__Proteobacteria.c__Epsilonproteobacteria.o__Campylobacterales. f__Campylobacteraceae**	24	3.973866	0.015949
k__Bacteria.p__Proteobacteria.c__Epsilonproteobacteria.o__Campylobacterales.f__Helicobacteraceae	24	4.244299	0.002041
**k__Bacteria.p__Proteobacteria.c__Epsilonproteobacteria.o__Campylobacterales.f__Helicobacteraceae. g__Flexispira**	24	4.23297	0.001257
k__Bacteria.p__Proteobacteria.c__Gammaproteobacteria	24	5.068394	1.87E-08
k__Bacteria.p__Proteobacteria.c__Gammaproteobacteria.o__Aeromonadales	0	2.55819	0.000249
k__Bacteria.p__Proteobacteria.c__Gammaproteobacteria.o__Aeromonadales.f__Succinivibrionaceae	0	2.540325	0.000249
k__Bacteria.p__Proteobacteria.c__Gammaproteobacteria.o__Aeromonadales.f__Succinivibrionaceae. g__Anaerobiospirillum	48	2.551319	0.000183
k__Bacteria.p__Proteobacteria.c__Gammaproteobacteria.o__Aeromonadales.f__Succinivibrionaceae. g__Succinivibrio	0	2.64469	0.03253
k__Bacteria.p__Proteobacteria.c__Gammaproteobacteria.o__Enterobacteriales	24	4.871518	9.6E-07
k__Bacteria.p__Proteobacteria.c__Gammaproteobacteria.o__Enterobacteriales.f__Enterobacteriaceae	24	4.871518	9.6E-07
**k__Bacteria.p__Proteobacteria.c__Gammaproteobacteria.o__Enterobacteriales.f__Enterobacteriaceae. g__**	24	4.476878	2.36E-06
**k__Bacteria.p__Proteobacteria.c__Gammaproteobacteria.o__Enterobacteriales.f__Enterobacteriaceae. g__Escherichia**	48	4.634761	1.91E-06
k__Bacteria.p__Proteobacteria.c__Gammaproteobacteria.o__Pasteurellales	24	4.630909	6.76E-09
k__Bacteria.p__Proteobacteria.c__Gammaproteobacteria.o__Pasteurellales.f__Pasteurellaceae	24	4.630909	6.76E-09
**k__Bacteria.p__Proteobacteria.c__Gammaproteobacteria.o__Pasteurellales.f__Pasteurellaceae. g__Actinobacillus**	24	4.332573	9.38E-06
**k__Bacteria.p__Proteobacteria.c__Gammaproteobacteria.o__Pasteurellales.f__Pasteurellaceae. g__Pasteurella**	48	4.366761	6.39E-07
k__Bacteria.p__Proteobacteria.c__Gammaproteobacteria.o__Pseudomonadales	48	3.159867	0.001144
k__Bacteria.p__Proteobacteria.c__Gammaproteobacteria.o__Pseudomonadales.f__Moraxellaceae	48	3.16189	0.001144
k__Bacteria.p__Proteobacteria.c__Gammaproteobacteria.o__Pseudomonadales.f__Moraxellaceae. g__Moraxella	48	3.161041	0.001144
k__Bacteria.p__Spirochaetes.c__Spirochaetes.o__Sphaerochaetales	0	3.816151	3.48E-06
k__Bacteria.p__Spirochaetes.c__Spirochaetes.o__Sphaerochaetales.f__Sphaerochaetaceae	0	3.816151	3.48E-06
k__Bacteria.p__Spirochaetes.c__Spirochaetes.o__Sphaerochaetales.f__Sphaerochaetaceae.__	0	2.825654	0.049112
**k__Bacteria.p__Spirochaetes.c__Spirochaetes.o__Sphaerochaetales.f__Sphaerochaetaceae. g__Sphaerochaeta**	0	3.812455	3.89E-06
k__Bacteria.p__Spirochaetes.c__Spirochaetes.o__Sphaerochaetales.f__Sphaerochaetaceae.g__wall_less	0	2.752892	0.022007

k__Bacteria.p__Synergistetes	0	2.412994	7.98E-07
k__Bacteria.p__Synergistetes.c__Synergistia	0	2.40993	7.98E-07
k__Bacteria.p__Synergistetes.c__Synergistia.o__Synergistales	0	2.413229	7.98E-07
k__Bacteria.p__Synergistetes.c__Synergistia.o__Synergistales.f__Dethiosulfovibrionaceae	0	2.481697	1.02E-06
k__Bacteria.p__Synergistetes.c__Synergistia.o__Synergistales.f__Dethiosulfovibrionaceae.<br>g__Pyramidobacter	0	2.473021	1.02E-06
k__Bacteria.p__Tenericutes	0	3.774633	1.86E-08
k__Bacteria.p__Tenericutes.c__Mollicutes	0	3.764449	1.04E-08
k__Bacteria.p__Tenericutes.c__Mollicutes.o__Acholeplasmatales	0	2.42373	0.027648
k__Bacteria.p__Tenericutes.c__Mollicutes.o__Acholeplasmatales.f__	0	2.387018	0.027648
k__Bacteria.p__Tenericutes.c__Mollicutes.o__Acholeplasmatales.f__.g__	0	2.385963	0.027648
k__Bacteria.p__Tenericutes.c__Mollicutes.o__Anaeroplasmatales	0	3.348045	7.57E-11
k__Bacteria.p__Tenericutes.c__Mollicutes.o__Anaeroplasmatales.f__Anaeroplasmataceae	0	3.348113	7.57E-11
k__Bacteria.p__Tenericutes.c__Mollicutes.o__Anaeroplasmatales.f__Anaeroplasmataceae.g__	0	3.054776	4.64E-09
k__Bacteria.p__Tenericutes.c__Mollicutes.o__Anaeroplasmatales.f__Anaeroplasmataceae.g__Anaeroplasma	0	3.052206	6.01E-10
k__Bacteria.p__Tenericutes.c__Mollicutes.o__Mycoplasmatales	0	2.391384	3E-05
k__Bacteria.p__Tenericutes.c__Mollicutes.o__Mycoplasmatales.f__Mycoplasmataceae	0	2.420854	3E-05
k__Bacteria.p__Tenericutes.c__Mollicutes.o__Mycoplasmatales.f__Mycoplasmataceae.g__Mycoplasma	0	2.369909	3E-05
k__Bacteria.p__Tenericutes.c__Mollicutes.o__RF39	0	3.537555	3E-07
k__Bacteria.p__Tenericutes.c__Mollicutes.o__RF39.f__	0	3.537555	3E-07
**k__Bacteria.p__Tenericutes.c__Mollicutes.o__RF39.f__.g__**	0	3.537555	3E-07
k__Bacteria.p__TM7	0	2.683579	1.69E-08
k__Bacteria.p__TM7.c__TM7_3	0	2.683579	1.69E-08
k__Bacteria.p__TM7.c__TM7_3.o__CW040	0	2.683579	1.69E-08
k__Bacteria.p__TM7.c__TM7_3.o__CW040.f__F16	0	2.683579	1.69E-08
k__Bacteria.p__TM7.c__TM7_3.o__CW040.f__F16.g__	0	2.683579	1.69E-08
k__Bacteria.p__Verrucomicrobia	0	2.727141	1.85E-09
k__Bacteria.p__Verrucomicrobia.c__Opitutae	0	3.197752	0.018684
k__Bacteria.p__Verrucomicrobia.c__Opitutae.o___Cerasicoccales_	0	3.236852	0.018684
k__Bacteria.p__Verrucomicrobia.c__Opitutae.o___Cerasicoccales_.f___Cerasicoccaceae_	0	3.215445	0.018684
k__Bacteria.p__Verrucomicrobia.c__Opitutae.o___Cerasicoccales_.f___Cerasicoccaceae_.g__	0	3.209237	0.018684
k__Bacteria.p__Verrucomicrobia.c__Verruco_5	0	2.489719	3.44E-10
k__Bacteria.p__Verrucomicrobia.c__Verruco_5.o__WCHB1_41	0	2.492531	3.44E-10
k__Bacteria.p__Verrucomicrobia.c__Verruco_5.o__WCHB1_41.f__RFP12	0	2.488791	1.34E-10
k__Bacteria.p__Verrucomicrobia.c__Verruco_5.o__WCHB1_41.f__RFP12.g__	0	2.483573	1.34E-10
k__Bacteria.p__Verrucomicrobia.c__Verrucomicrobiae	0	2.460908	3.25E-06

k__Bacteria.p__Verrucomicrobia.c__Verrucomicrobiae.o__Verrucomicrobiales	0	2.460348	3.25E-06
k__Bacteria.p__Verrucomicrobia.c__Verrucomicrobiae.o__Verrucomicrobiales.f__Verrucomicrobiaceae	0	2.460722	3.25E-06
k__Bacteria.p__Verrucomicrobia.c__Verrucomicrobiae.o__Verrucomicrobiales.f__Verrucomicrobiaceae.g__Akkermansia	0	2.460203	3.25E-06
k__Bacteria.p__WPS_2	0	2.587993	7.86E-05
k__Bacteria.p__WPS_2.c__	0	2.589441	7.86E-05
k__Bacteria.p__WPS_2.c__.o__	0	2.586975	7.86E-05
k__Bacteria.p__WPS_2.c__.o__.f__	0	2.587791	7.86E-05
k__Bacteria.p__WPS_2.c__.o__.f__.g__	0	2.59418	7.86E-05

Table S3. Differential abundance across resuscitation identified by LEfSe. Differentially abundant clades were defined as $p < 0.05$ and LDA > 2.

Clade	Resusc.	Log_{10} LDA	*p*-value
k__Bacteria.p__Actinobacteria.c__Actinobacteria.o__Actinomycetales.f__Actinomycetaceae.g__Arcanobacterium	Cr	3.724628	0.025977
k__Bacteria.p__Actinobacteria.c__Actinobacteria.o__Actinomycetales.f__Actinomycetaceae.g__Mobiluncus	Co	3.180036	0.015012
k__Bacteria.p__Actinobacteria.c__Actinobacteria.o__Actinomycetales.f__Actinomycetaceae.g__Trueperella	Co	3.340407	0.010636
k__Bacteria.p__Bacteroidetes.c__Bacteroidia.o__Bacteroidales.f__	MB	4.49443	0.014788
k__Bacteria.p__Bacteroidetes.c__Bacteroidia.o__Bacteroidales.f__.g__	MB	4.49443	0.014788
k__Bacteria.p__Bacteroidetes.c__Bacteroidia.o__Bacteroidales.f__Prevotellaceae.__	MB	2.151348	0.008116
k__Bacteria.p__Firmicutes.c__Bacilli.o__Lactobacillales.f__Streptococcaceae	Co	4.120484	0.009545
k__Bacteria.p__Firmicutes.c__Bacilli.o__Lactobacillales.f__Streptococcaceae.g__Streptococcus	Co	4.120484	0.009545
k__Bacteria.p__Firmicutes.c__Bacilli.o__Turicibacterales	Co	3.554226	0.023796
k__Bacteria.p__Firmicutes.c__Bacilli.o__Turicibacterales.f__Turicibacteraceae	Co	3.671839	0.023796
k__Bacteria.p__Firmicutes.c__Bacilli.o__Turicibacterales.f__Turicibacteraceae.g__Turicibacter	Co	3.341864	0.023796
k__Bacteria.p__Firmicutes.c__Clostridia.o__Clostridiales.__	MB	3.836899	0.021349
k__Bacteria.p__Firmicutes.c__Clostridia.o__Clostridiales.__.__	MB	3.836899	0.021349
k__Bacteria.p__Firmicutes.c__Clostridia.o__Clostridiales.f___Tissierellaceae_.g__Gallicola	Co	2.049709	0.008773
k__Bacteria.p__Firmicutes.c__Clostridia.o__Clostridiales.f__Clostridiaceae.g__Sarcina	Co	2.156812	0.010556
k__Bacteria.p__Firmicutes.c__Clostridia.o__Clostridiales.f__Clostridiaceae.g__SMB53	Co	2.763974	0.009133
k__Bacteria.p__Firmicutes.c__Clostridia.o__Clostridiales.f__Lachnospiraceae.g___Ruminococcus_	Cr	2.310848	0.019491
k__Bacteria.p__Firmicutes.c__Clostridia.o__Clostridiales.f__Peptococcaceae.g__Peptococcus	Co	2.246513	0.000749
k__Bacteria.p__Firmicutes.c__Clostridia.o__Clostridiales.f__Peptostreptococcaceae	Co	2.673343	0.049014
k__Bacteria.p__Firmicutes.c__Clostridia.o__Clostridiales.f__Ruminococcaceae.g__Subdoligranulum	Co	2.444097	0.033417
k__Bacteria.p__Firmicutes.c__Clostridia.o__Clostridiales.f__Veillonellaceae	Co	3.588208	0.02958
k__Bacteria.p__Firmicutes.c__Erysipelotrichi.o__Erysipelotrichales.f__Erysipelotrichaceae.g__Clostridium	Co	2.470861	0.012487
k__Bacteria.p__Proteobacteria.c__Alphaproteobacteria.__	MB	2.830856	0.038477
k__Bacteria.p__Proteobacteria.c__Alphaproteobacteria.__.__	MB	2.692976	0.038477
k__Bacteria.p__Proteobacteria.c__Alphaproteobacteria.__.__.__	MB	2.781238	0.038477
k__Bacteria.p__Proteobacteria.c__Betaproteobacteria.o__Burkholderiales.f__Comamonadaceae	MB	3.167328	0.000157

k__Bacteria.p__Proteobacteria.c__Betaproteobacteria.o__Burkholderiales.f__Comamonadaceae.g__Delftia	MB	3.159569	0.000157
k__Bacteria.p__Proteobacteria.c__Epsilonproteobacteria.o__Campylobacterales.f__Campylobacteraceae.g__Campylobacter	Cr	4.189113	0.02345
k__Bacteria.p__Proteobacteria.c__Epsilonproteobacteria.o__Campylobacterales.f__Helicobacteraceae.__	MB	3.357528	0.038834
k__Bacteria.p__Proteobacteria.c__Gammaproteobacteria.o__Pasteurellales.f__Pasteurellaceae.g__Actinobacillus	Co	4.175826	0.049957
k__Bacteria.p__Verrucomicrobia.c__Verruco_5.o__WCHB1_41.__	MB	3.155323	0.024088
k__Bacteria.p__Verrucomicrobia.c__Verruco_5.o__WCHB1_41.__.__	MB	3.15263	0.024088
k__Bacteria.p__Verrucomicrobia.c__Verruco_5.o__WCHB1_41.f__WCHB1_25	MB	3.048606	0.00328
k__Bacteria.p__Verrucomicrobia.c__Verruco_5.o__WCHB1_41.f__WCHB1_25.g__	MB	3.050631	0.00328

Table S4. Pathways perturbed by limited volume resuscitation. Blank cells indicate that the pathway was not identified as significant ($q > 0.05$). Pathways that were perturbed by both LV-Co and LV-Cr resuscitation are indicated by *.

Pathway	LV- Cr *q* value	LV- Co *q* value
*ssc00190 Oxidative phosphorylation	5.83E-06	3.41E-10
*ssc04152 AMPK signaling pathway	5.83E-06	5.71E-05
*ssc04141 Protein processing in endoplasmic reticulum	6.42E-06	3.78E-06
*ssc04146 Peroxisome	7.58E-05	6.01E-04
*ssc04140 Autophagy - animal	7.58E-05	6.78E-04
*ssc01200 Carbon metabolism	3.36E-04	4.52E-04
*ssc00260 Glycine, serine and threonine metabolism	7.71E-04	3.25E-03
*ssc00982 Drug metabolism - cytochrome P450	1.18E-03	3.72E-04
*ssc03010 Ribosome	1.79E-03	5.71E-05
*ssc04145 Phagosome	1.26E-03	6.65E-04
*ssc00900 Terpenoid backbone biosynthesis	1.50E-03	2.02E-02
*ssc03320 PPAR signaling pathway	1.79E-03	6.45E-03
*ssc00310 Lysine degradation	1.79E-03	1.61E-03
*ssc00100 Steroid biosynthesis	1.92E-03	9.17E-03
*ssc04621 NOD-like receptor signaling pathway	8.51E-03	6.01E-04
*ssc04976 Bile secretion	3.17E-03	1.87E-02
*ssc01212 Fatty acid metabolism	3.86E-03	7.58E-03
*ssc04666 Fc gamma R-mediated phagocytosis	5.76E-03	2.41E-02
*ssc04150 mTOR signaling pathway	5.91E-03	1.74E-02
*ssc00270 Cysteine and methionine metabolism	1.20E-02	4.89E-03
*ssc04920 Adipocytokine signaling pathway	8.28E-03	4.93E-03
*ssc03018 RNA degradation	6.30E-03	9.17E-03
*ssc00830 Retinol metabolism	1.12E-02	5.08E-03
*ssc03040 Spliceosome	3.27E-02	5.90E-03
*ssc00140 Steroid hormone biosynthesis	3.48E-02	6.45E-03
*ssc04142 Lysosome	9.99E-03	1.36E-02
*ssc00280 Valine, leucine and isoleucine degradation	3.48E-02	6.45E-03
*ssc04217 Necroptosis	1.12E-02	1.82E-03
*ssc00040 Pentose and glucuronate interconversions	1.23E-02	1.18E-02
*ssc04530 Tight junction	3.88E-02	1.20E-02
*ssc04216 Ferroptosis	2.01E-02	1.69E-02
*ssc00053 Ascorbate and aldarate metabolism	2.54E-02	1.87E-02
*ssc03015 mRNA surveillance pathway	3.28E-02	1.69E-02
ssc00513 Various types of N-glycan biosynthesis	1.86E-02	
ssc04659 Th17 cell differentiation	1.89E-02	
ssc04218 Cellular senescence	4.09E-02	
ssc03008 Ribosome biogenesis in eukaryotes	4.12E-02	

ssc04211 Longevity regulating pathway	1.26E-03	
ssc04922 Glucagon signaling pathway	1.05E-04	
ssc04910 Insulin signaling pathway	7.19E-04	
ssc00220 Arginine biosynthesis	1.12E-02	
ssc03050 Proteasome	2.30E-02	
ssc04620 Toll-like receptor signaling pathway	2.51E-02	
ssc04068 FoxO signaling pathway	2.86E-02	
ssc04520 Adherens junction	2.86E-02	
ssc04066 HIF-1 signaling pathway	2.86E-02	
ssc04120 Ubiquitin mediated proteolysis		1.36E-02
ssc00380 Tryptophan metabolism		1.87E-02
ssc00480 Glutathione metabolism		2.41E-02
ssc03013 RNA transport		2.82E-02
ssc04919 Thyroid hormone signaling pathway		2.90E-02
ssc04064 NF-kappa B signaling pathway		3.60E-02
ssc00630 Glyoxylate and dicarboxylate metabolism		6.45E-03
ssc01230 Biosynthesis of amino acids		1.32E-03
ssc00071 Fatty acid degradation		1.01E-02
ssc03060 Protein export		1.87E-02
ssc04622 RIG-I-like receptor signaling pathway		1.87E-02
ssc00650 Butanoate metabolism		1.87E-02

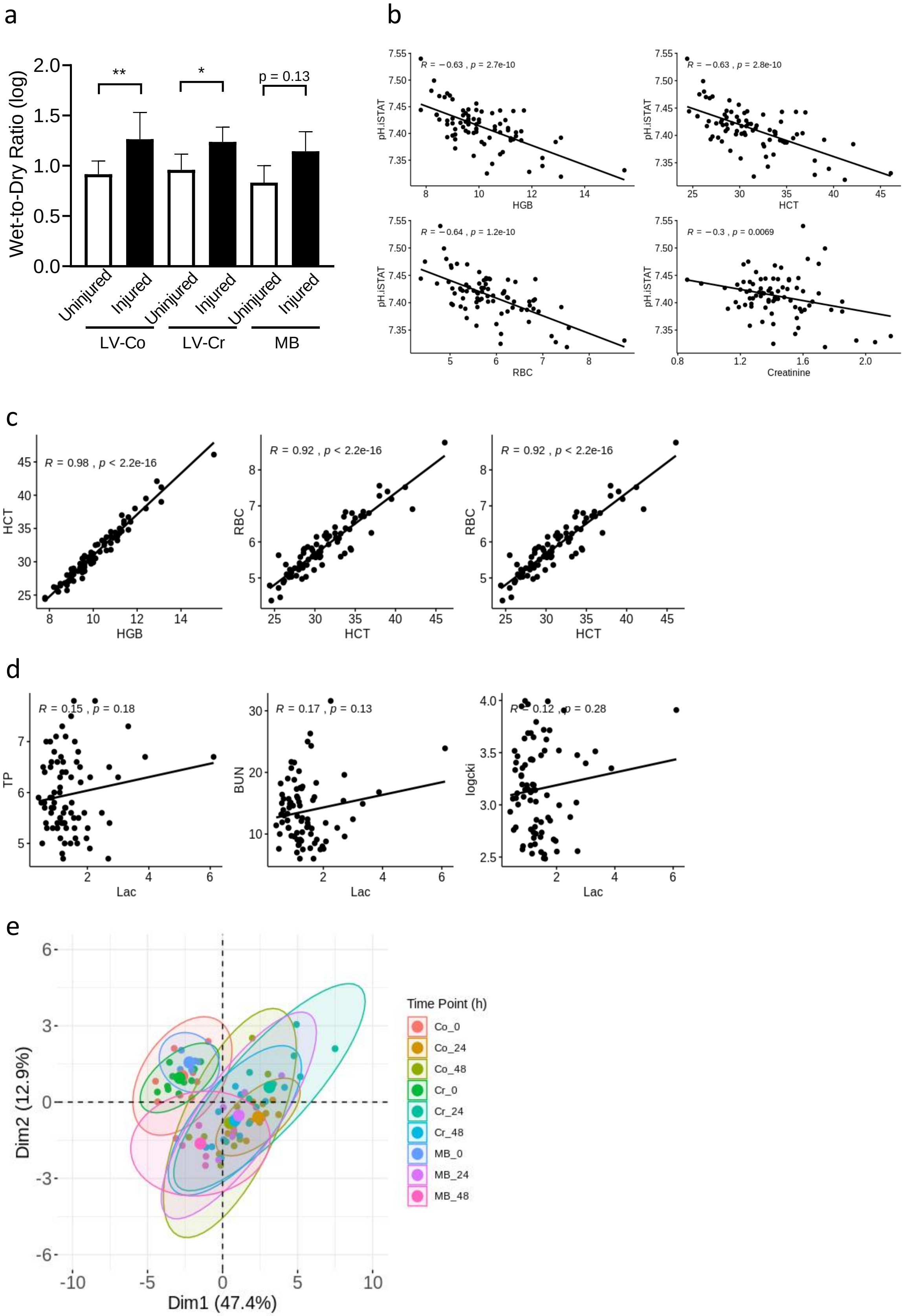

a
b
c
d
e
Wet-to-Dry Ratio (log)
**
*
p = 0.13
Uninjured
Injured
LV-Co
LV-Cr
MB
R = 0.98 , p < 2.2e-16
R = 0.92 , p < 2.2e-16
R = 0.15 , p = 0.18
R = 0.17 , p = 0.13
R = 0.12 , p = 0.28
Time Point (h)
Co_0
Co_24
Co_48
Cr_0
Cr_24
Cr_48
MB_0
MB_24
MB_48
Dim1 (47.4%)
Dim2 (12.9%)

Figure S1. Additional analyses of limited-volume resuscitation impact on burn pathophysiology. (**a**) Wet-to-dry weight ratio, in uninjured and burn-injured skin 48 h after injury. *P* values were calculated by two-way ANOVA with Holm family-wise error rate correction at $\alpha = 0.05$. *p<0.05, **p<0.01, ***p<0.001. (**b-d**) Regression analysis of clinical biomarkers confirming associations identified by PCA. (**e**) Stratification of the clinical biomarker dataset for each combination of resuscitation strategy and injury time-point. MB (n = 6 pigs), LV-Co (n = 12 pigs), and LV-Cr (n = 12 pigs).

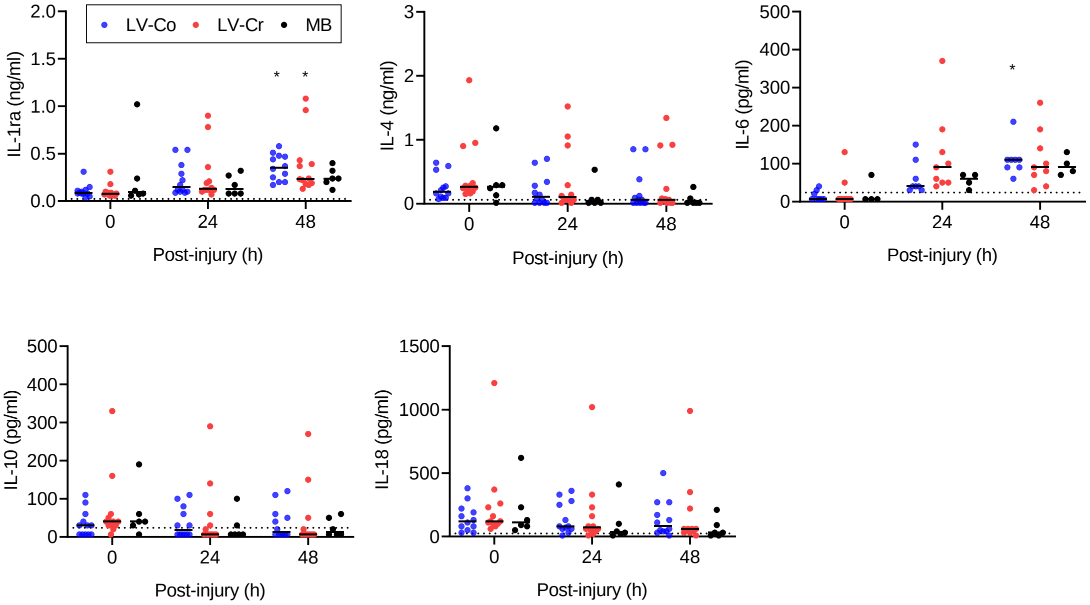

Figure S2. Changes in plasma cytokines after burn and resuscitation. Plasma cytokines were quantified by multiplex bead assay. Cytokines which were below the limit of quantification (GM-CSF, IFNγ, IL-1β) for ≥50% of samples were excluded from analysis. Horizontal black bars and dashed lines depict the median and limit of quantification, respectively. Each data point represents an individual sample. *: $p < 0.05$ compared with 0 h calculated by Wilcoxon rank-sum test with Holm family-wise error rate correction at $\alpha = 0.05$.

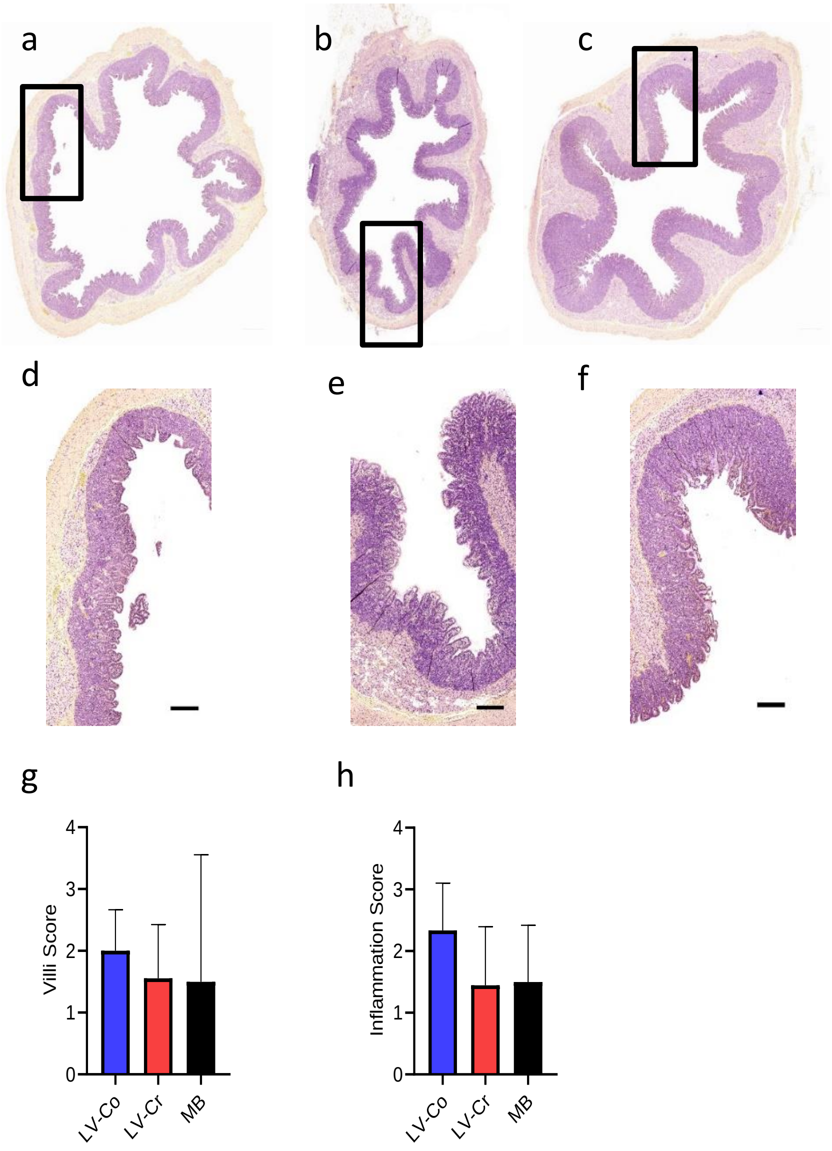

Figure S3. Villus architecture and inflammation after burn and resuscitation. (**a-c**) Representative sections of the duodenum from (**a**) LV-Co, (**b**) LV-Cr, and (**c**) MB pigs were stained with a Modified Gram stain. (**d-f**) Magnification of areas demarcated by black boxes in panels a-c, respectively. Scale bar is 500 µm. Images were acquired with the ZEN2 software v2.0. Tissue sections were evaluated and scored for (**g**) villi architecture and (**h**) inflammation. Bars show the mean and 95% CI. Wilcoxon rank-sum testing did not identify any significant difference among resuscitation strategies.

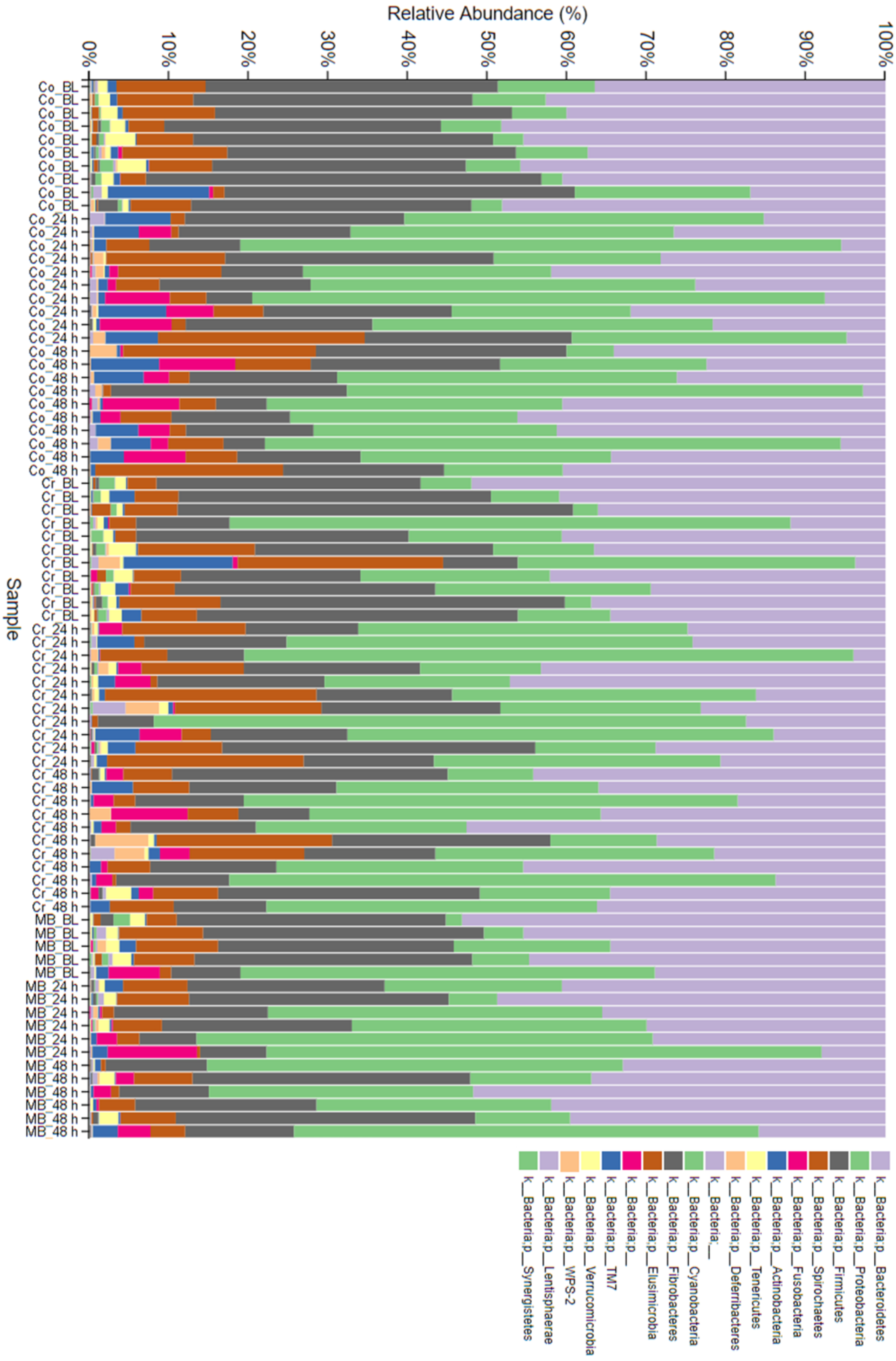
Relative Abundance (%)
0%
10%
20%
30%
40%
50%
60%
70%
80%
90%
100%
Sample
Co_BL
Co_24 h
Co_48 h
Cr_BL
Cr_24 h
Cr_48 h
MB_BL
MB_24 h
MB_48 h
K__Bacteria;p__Bacteroidetes
K__Bacteria;p__Proteobacteria
K__Bacteria;p__Firmicutes
K__Bacteria;p__Spirochaetes
K__Bacteria;p__Fusobacteria
K__Bacteria;p__Actinobacteria
K__Bacteria;p__Tenericutes
K__Bacteria;p__Deferribacteres
K__Bacteria;__
K__Bacteria;p__Cyanobacteria
K__Bacteria;p__Fibrobacteres
K__Bacteria;p__Elusimicrobia
K__Bacteria;p__
K__Bacteria;p__TM7
K__Bacteria;p__Verrucomicrobia
K__Bacteria;p__WPS-2
K__Bacteria;p__Lentisphaerae
K__Bacteria;p__Synergistetes

Figure S4. Changes in bacterial composition after burn and resuscitation. Taxonomic bar plot at the phylum level showing bacterial communities from rectal swabs analyzed by 16S rRNA gene sequencing at baseline (BL), 24 h, and 48 h after burn injury and resuscitation. Each bar depicts the relative abundance for an individual sample. Sample designations indicate the type of resuscitation fluid that was administered (LV-Co, LV-Cr, or MB) and the sample time-point (BL, 24 h, 48h).

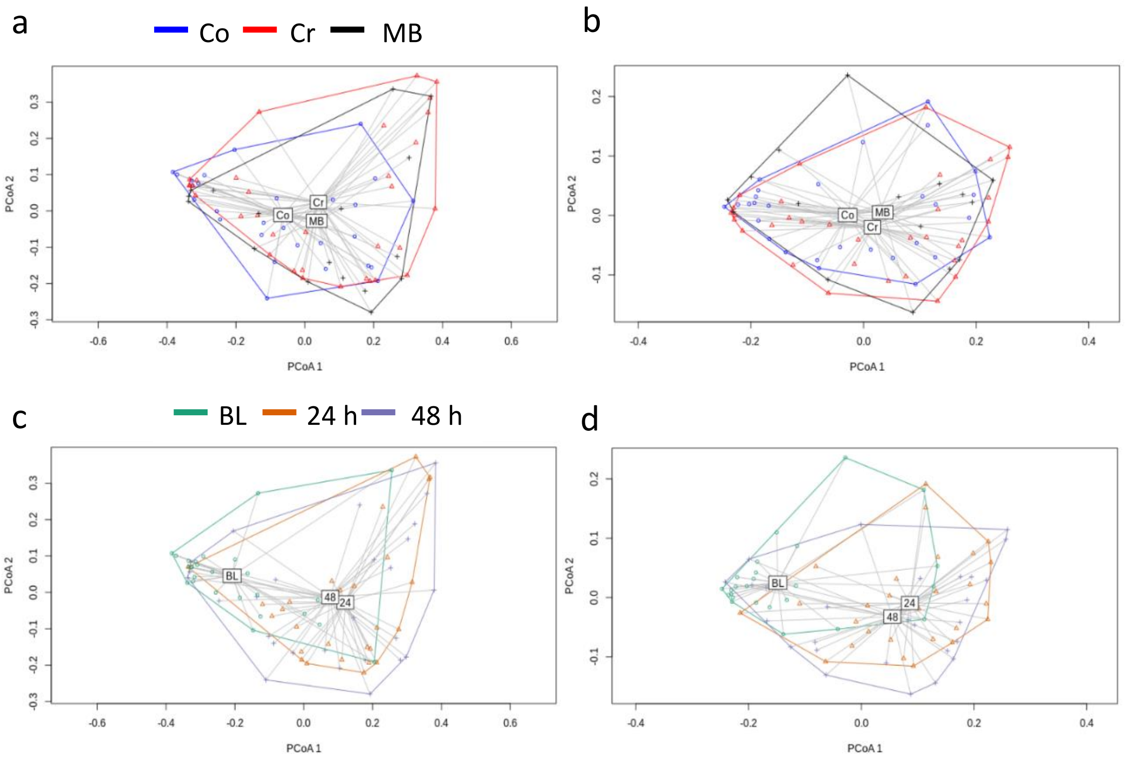

Figure S5. Within resuscitation-group dispersion of Bray-Curtis and generalized UniFrac using spatial medians. Dispersion of (**a**) Bray-Curtis and (**b**) generalized UniFrac grouped by resuscitation strategy, and (**c**) Bray-Curtis and (**d**) generalized UniFrac grouped by injury time. Plots were created with betadisper in vegan v2.5. LV-Co: n = 10, LV-Cr: n = 11, MB: n = 6.

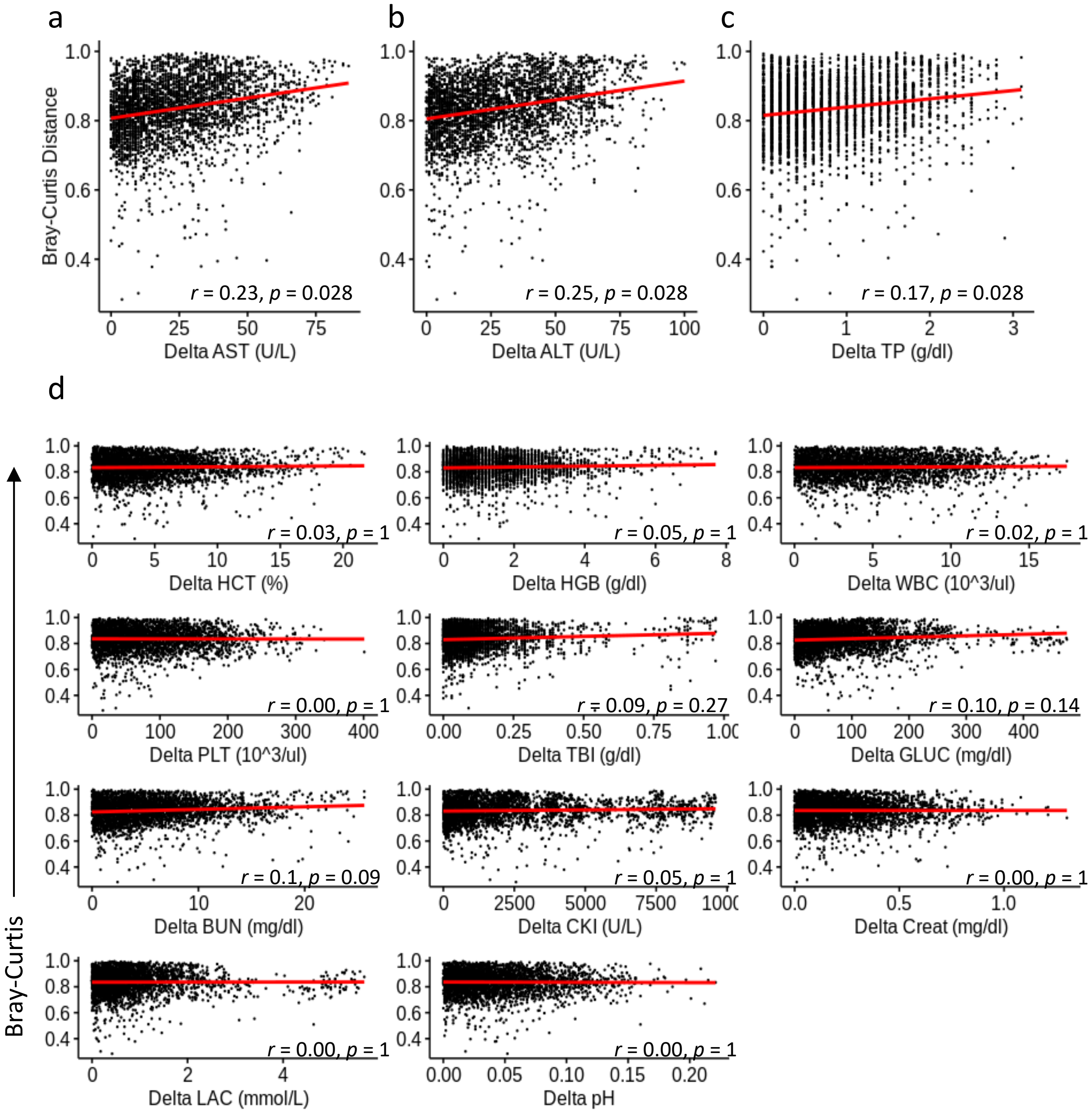

Figure S6. Correlation analyses of clinical biomarkers and Bray-Curtis. (**a-c**) Mantel correlation analysis between Bray-Curtis and (**a**) AST, (**b**) ALT, and (**c**) TP. (**d**) Clinical biomarkers that were not significantly correlated with Bray-Curtis. Correlation coefficients were calculated by Mantel test and indicated *p* values were Holm corrected for family-wise error rate at α = 0.05. A family of tests was defined as those shown in Figure 5a-c, Figure S5, and Figure S6. MB (n = 6 pigs), LV-Co (n = 10 pigs), and LV-Cr (n = 11 pigs).

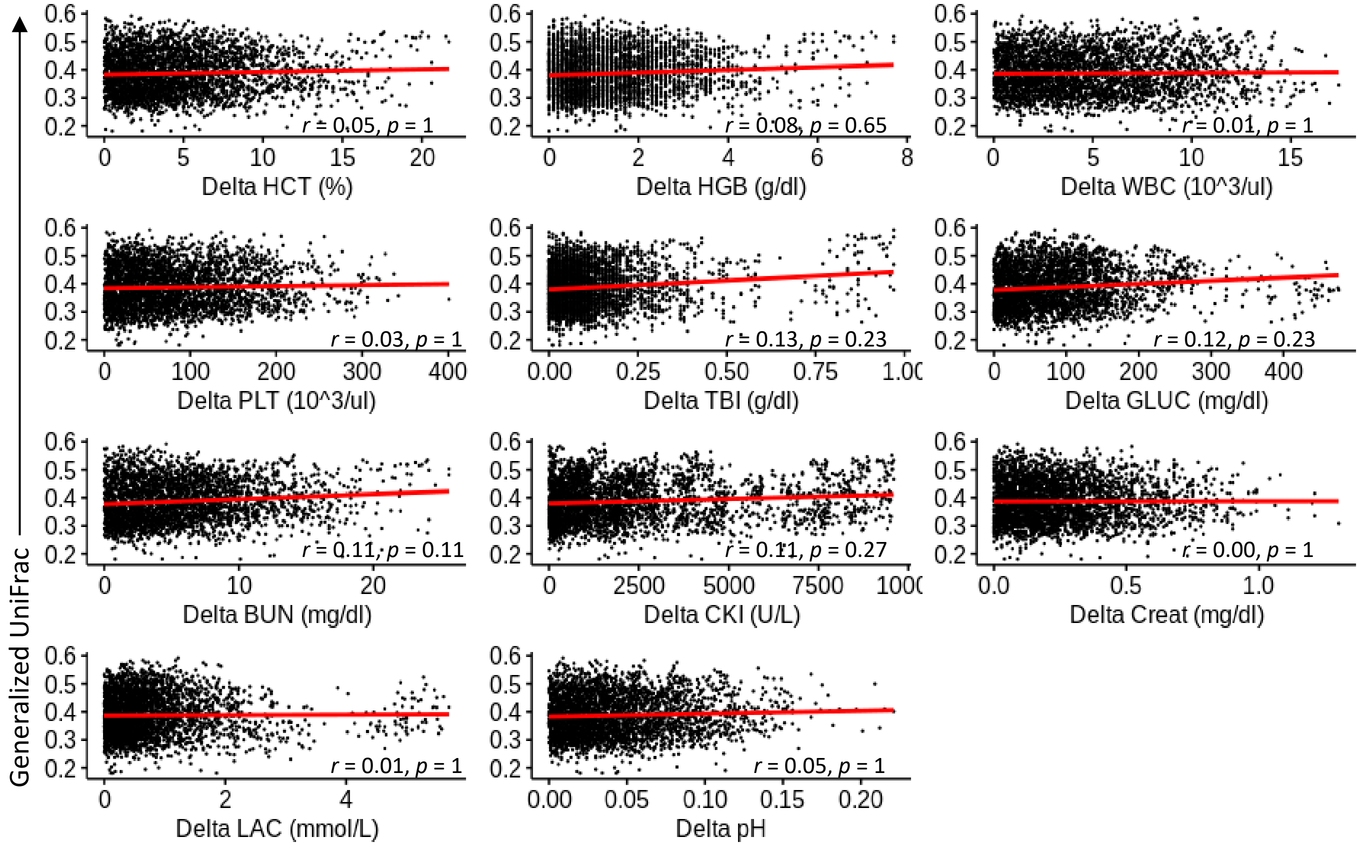

Figure S7. Clinical biomarkers not significantly correlated with generalized UniFrac. Correlation coefficients were calculated by Mantel test and indicated *p* values were Holm corrected for family-wise error rate at α = 0.05. A family of tests was defined as those shown in Figure 5a-c, Figure S5, and Figure S6. MB (n = 6 pigs), LV-Co (n = 10 pigs), and LV-Cr (n = 11 pigs).

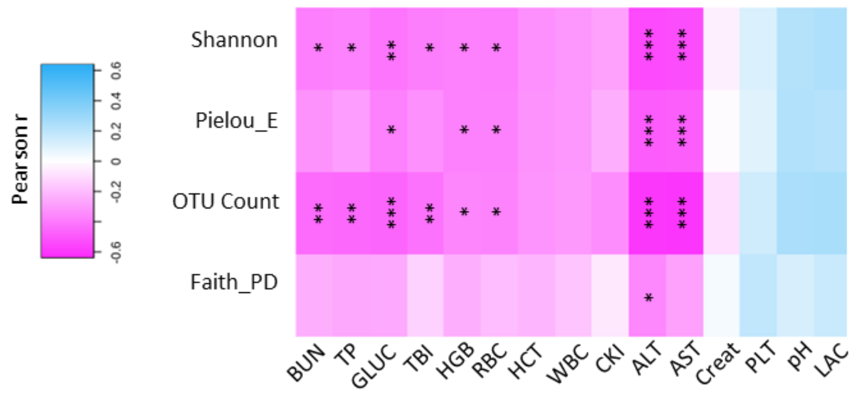

Figure S8. Associations of alpha diversities and clinical biomarkers. Strength of Pearson correlation coefficients were visualized by heatmap. *p* values were corrected for multiple comparisons with Holm family-wise error rate at α = 0.05. *p<0.05, **p<0.01, ***p<0.001. BUN: blood urea nitrogen, TP: total protein, GLUC: glucose, TBI: total bilirubin, HGB: hemoglobin, RBC: red blood cell count, HCT: hematocrit, WBC: white blood cell count, CKI: creatine kinase, ALT: alanine aminotransferase, AST: aspartate aminotransferase, Creat: creatinine, PLT: platelet count, LAC: lactate. MB (n = 6 pigs), LV-Co (n = 10 pigs), and LV-Cr (n = 11 pigs).

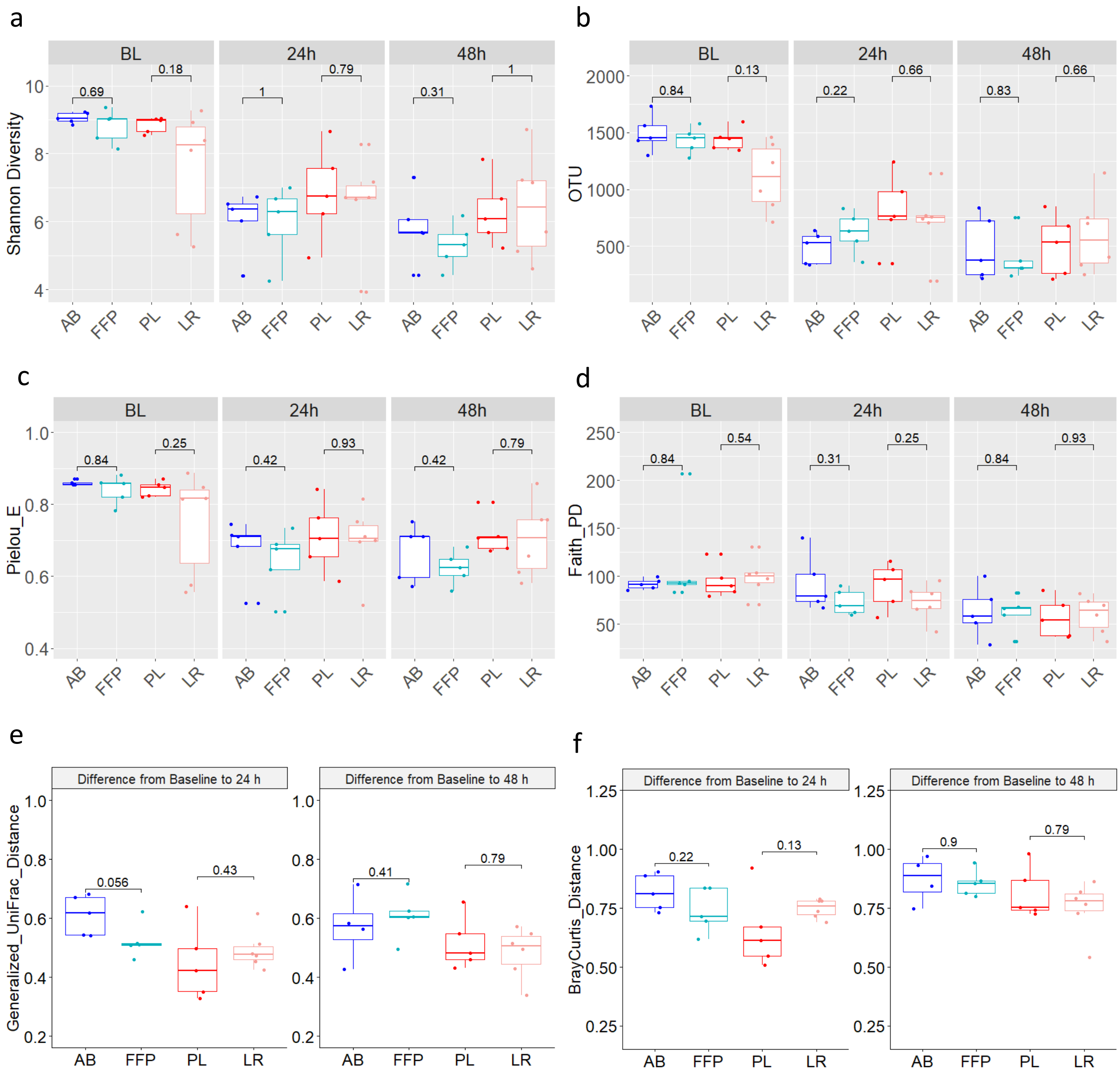

Figure S9. Bacterial diversity between AB and FFP, and LR and PL. (**a-d**) Alpha and (**e, f**) beta diversity metrics were evaluated for differences within LV-Co and LV-Cr groups. Each boxplot shows the median along with the first and third quartiles. Whiskers depict the standard error and data points in the boxplots represent an individual sample. Numbers above brackets indicate Wilcoxon test p-value.
